# TCR signal strength controls thymic differentiation of iNKT cell subsets

**DOI:** 10.1038/s41467-018-05026-6

**Published:** 2018-07-09

**Authors:** Kathryn D. Tuttle, S. Harsha Krovi, Jingjing Zhang, Romain Bedel, Laura Harmacek, Lisa K. Peterson, Leonard L. Dragone, Adam Lefferts, Catherine Halluszczak, Kent Riemondy, Jay R. Hesselberth, Anjana Rao, Brian P. O’Connor, Philippa Marrack, James Scott-Browne, Laurent Gapin

**Affiliations:** 1 Department of Immunology and Microbiology, University of Colorado Anschutz Medical Campus, 12800 E. 19th Ave, Aurora, CO 80045 USA; 2 Department of Biomedical Research, National Jewish Health, 1400 Jackson Street, Denver, CO 80206 USA; 3 Center for Genes, Environment, and Health, Department of Biomedical Research, National Jewish Health, 1400 Jackson Street, Denver, CO 80206 USA; 4 Department of Pediatrics, National Jewish Health, 1400 Jackson Street, Denver, 80206 CO USA; 5 RNA Bioscience Initiative, University of Colorado School of Medicine, 12800 E. 19th Ave, Aurora, 80045 CO USA; 6 Department of Biochemistry & Molecular Genetics, University of Colorado School of Medicine, 12800 E. 19th Ave, Aurora, CO 80045 USA; 7 La Jolla Institute, 9420 Athena Cir, La Jolla, 92037 CA USA; 8 Sanford Consortium for Regenerative Medicine, 2880 Torrey Pines Scenic Dr, La Jolla, CA 92037 USA; 9 University of California San Diego, 9500 Gilman Drive, La Jolla, CA 92093 USA; 10 Department of Medicine, University of Colorado Anschutz Medical Campus, 12800 E. 19th Ave, Aurora, CO 80045 USA; 11 Present Address: Department of Oncology, University of Lausanne, Chemin des Boveresses 155, Epalinges, 1066 Switzerland; 12 ARUP Laboratories, Institute of Clinical and Experimental Pathology, 500 Chipeta Way, Salt Lake City, 84108 UT Switzerland; 13 Department of Pathology, University of Utah, 30N 1900E, Salt Lake City, 84132 UT USA; 14 Present Address: Merck Research Laboratories, San Francisco, CA USA

## Abstract

During development in the thymus, invariant natural killer T (iNKT) cells commit to one of three major functionally different subsets, iNKT1, iNKT2, and iNKT17. Here, we show that T cell antigen receptor (TCR) signal strength governs the development of iNKT cell subsets, with strong signaling promoting iNKT2 and iNKT17 development. Altering TCR diversity or signaling diminishes iNKT2 and iNKT17 cell subset development in a cell-intrinsic manner. Decreased TCR signaling affects the persistence of *Egr2* expression and the upregulation of PLZF. By genome-wide comparison of chromatin accessibility, we identify a subset of iNKT2-specific regulatory elements containing NFAT and Egr binding motifs that is less accessible in iNKT2 cells that develop from reduced TCR signaling. These data suggest that variable TCR signaling modulates regulatory element activity at NFAT and Egr binding sites exerting a determinative influence on the dynamics of gene enhancer accessibility and the developmental fate of iNKT cells.

## Abstract (web summary)

Invariant natural killer T (iNKT) cells can be subsetted by their cytokine profiles, but how they develop in the thymus is unclear. Here the authors show, by analysing mice carrying mutant *Zap70* genes, that T cell receptor signaling strength induces epigenetic changes of genes to modulate iNKT lineages.

## Introduction

iNKT cells recognize lipid antigens presented by CD1d, a non-polymorphic major histocompatibility complex (MHC) class I-like antigen-presenting molecule^1^. These cells use a semi-invariant TCR made up mostly of a single invariant TCRα chain (Vα14-Jα18 in mice, Vα24-Jα18 in humans) with certain TCRβ chains (Vβ8.2, Vβ7, or Vβ2 in mice, Vβ11 in humans) to engage CD1d. In the thymus, iNKT cells develop into three major terminally differentiated and functionally distinct iNKT cell subsets^2,3^. iNKT1 cells express the transcription factor T-bet and secrete predominantly IFNγ; iNKT2 cells express high levels of the GATA3 and promyelocytic leukaemia zinc finger (PLZF) transcription factors and secrete IL-4 and IL-13; iNKT17 have intermediate levels of PLZF, are positive for RAR-related orphan receptor gamma (Rorγt) expression, and secrete IL-17. Importantly, the relative distribution of the three thymic iNKT subsets varies in different mouse strains and affects the phenotype and activation status of surrounding cells.

Unlike most T cells that leave the thymus to populate the peripheral immune organs, some mature iNKT cells are retained in the thymus and become long-term thymic residents^4^, with potentially important functions. In particular, mature thymic iNKT2 cells produce IL-4 at steady state and thus affect the homeostasis of thymic cell populations^2^. Indeed, IL-4 conditions CD8^+^ T cells to become “memory-like” and to express the transcription factor Eomesodermin^5^. These CD8 “memory-like” T cells have important roles in early defenses, particularly in situations of chronic viral infection^6,7^. Thymic steady-state IL-4 also drives the acquisition of an activated/memory-like phenotype by Foxp3^+^ regulatory T cells^8^, the production of chemokines by thymic dendritic cells^2^, the thymic exit of mature “conventional” T cells^9^ and also perhaps the commitment of early thymic progenitors to the T cell lineage^10^. Additionally, RANKL-expressing CD44^−^ thymic iNKT cells (which are preferentially enriched for iNKT2 and iNKT17 cells) regulate the differentiation of Aire^+^ MHC class II^+^ medullary thymic epithelial cells^11^ that are involved in clonal deletion of self-reactive T cells^12^ and Treg maturation^13^. Altogether, these results suggest that thymic iNKT cells, and particularly the relative subset representation, have fundamental roles in the composition of other thymic cell populations, both by modulating homeostasis and maturation status of these cells, and potentially in shaping the overall size and repertoire diversity of mature “conventional” T cells.

Development of iNKT cells diverges from that of conventional T cells primarily at the double-positive CD4^+^ CD8^+^ (DP) stage and requires TCR recognition of CD1d on DP cells, involving homotypic interactions across a DP–DP synapse where second signals are initiated by the engagement of homophilic receptors of the signaling lymphocytic-activation molecule (SLAM) family, Slamf1 (SLAM) and Slamf6 (Ly108). This signaling recruits the adaptor SLAM-associated protein (SAP) and the Src kinase Fyn, both of which are essential for the development of the iNKT cell lineage^3^. The TCR signals received by iNKT cell precursors during selection are associated with high expression of the Ras-^14^ and Ca^2+^-dependent transcription factors Egr1 and, especially, Egr2^15,16^. Interestingly, high expression levels of Egr2 in pre-selection DP thymocytes are potentiated by co-stimulation through Ly108^17,18^. Egr2 directly regulates the expression of several genes involved in the development of iNKT cells, including PLZF and CD122, one of the chains of the IL-15 receptor^15^. Egr2 is recruited to the promoter of *Zbtb16* (which encodes PLZF) after TCR engagement and co-stimulation with Ly108^15,17^. PLZF directs the acquisition of effector properties, such as the upregulation of CD44 and production of effector cytokines^19,20^. Expression of PLZF directs the acquisition of effector properties by binding and regulating T helper-specific transcription factor genes that in turn control T-helper-specific programs^19–21^. Several transcription factors and signaling molecules affect the lineage diversification of iNKT cell subsets. On the whole, however, the mechanisms that control these iNKT cell fate decisions during development remain poorly understood.

Here, we show that TCR signal strength governs the development of iNKT cell subsets in the thymus, with high signal strength being necessary for iNKT2 and iNKT17 development. The avidity of the iNKT TCR–CD1d interaction correlates with iNKT cell subset assignment and the expression of markers reflecting strength of signaling during selection, suggesting that differential TCR signal strength likely has a role in iNKT cell subset differentiation. Limiting the iNKT TCR repertoire diversity through the use of TCRβ transgenic mice and/or TCR signaling using mice with Zap70 mutations markedly impacts iNKT2 and iNKT17 cell subset development in a cell-intrinsic manner. Decreased TCR signaling affects the longevity of Egr2 expression and the upregulation of PLZF both in vitro and in vivo. Genome-wide chromatin accessibility analysis reveals subset-specific activity of regulatory elements associated with unique transcription factor binding site signatures. NFAT and Egr binding motifs are found preferentially enriched in chromatin regulatory regions specifically accessible in iNKT2 cells that are not accessible in iNKT2 cells that had developed with reduced TCR signaling. Altogether, these data suggest a model of iNKT cell subset development wherein variable TCR signaling induces changes in chromatin accessibility at NFAT and Egr binding sites that exert a determinative influence on the dynamics of gene enhancer accessibility that in turn determines the developmental fate of iNKT cells.

## Results

### Characteristics of thymic iNKT cell subsets

**Fig. 1:**
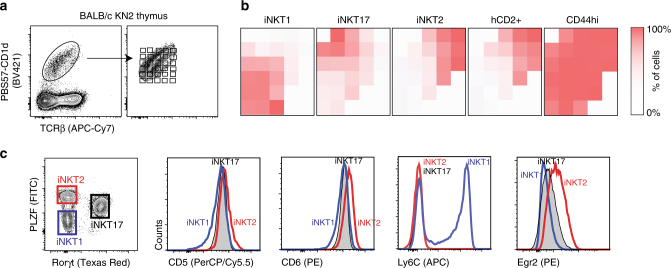
Thymic iNKT subsets have different avidities for the PBS57–CD1d tetramer that correlate with expression of surrogate markers reflective of strength of signaling during selection. **a** Cells from the thymus of BALB/c IL-4 reporter KN2 mice were stained with anti-TCRβ mAbs and PBS57–CD1d tetramers. iNKT cells were then electronically placed on a grid consisting of 30 gates, allowing for the analysis of iNKT cells expressing different TCR levels and/or different binding to the PBS57–CD1d tetramer for a given level of TCR. **b** The proportion of iNKT1 (PLZF^lo^, Rorγt^−^, Tbet^+^), iNKT17 (PLZF^int^, Rorγt^+^, Tbet^−^), iNKT2 (PLZF^hi^, Rorγt^–^, Tbet^–^, or hCD2^+^) as well as the proportion of CD44^hi^ cells in each gate was recorded and displayed as a heatmap. **c** Representative histograms for the expression of CD5, CD6, Ly6C, and Egr2 in each iNKT cell subset (as defined by the gating strategy shown on the left) is shown. Data are representative of *n* > 3 mice for each staining

We analyzed thymic iNKT cells from BALB/c mice in which the human CD2 gene was knocked-in into the IL-4 locus (KN2), thereby allowing for the identification by flow cytometry of IL-4-secreting iNKT2 cells directly ex vivo. iNKT cells, identified using PBS57–CD1d tetramer and TCRβ co-staining, were electronically placed on a grid consisting of 30 gates, allowing for the analysis of iNKT cells expressing different TCR levels and/or different binding to the PBS57–CD1d tetramer for a given level of TCR (Fig. 1a). Thus, each gate examined cells with different avidities for the antigen–CD1d complex. The proportion of each iNKT subset in each of the 30 different gates was calculated (Supplementary Fig 1) and displayed as a heatmap. Although not all gates contain the same number of cells, displaying the proportion of each iNKT cell subsets in the 30 gates revealed a direct correlation between the overall avidity of the TCR–PBS57–CD1d interaction and the three major iNKT cell subset phenotypes (Fig. 1b). iNKT1 cells express the lowest amount of TCR and are stained by the tetramer with the lowest intensity. iNKT17 cells express intermediate levels of TCR. However, for a given level of TCR expression, iNKT17 cells bind the tetramer more strongly than iNKT1 cells. Finally, iNKT2 cells express the highest TCR levels and stained more brightly with the PBS57–CD1d tetramer. This correlation is not observed with another marker of mature iNKT cells (CD44). These results demonstrate that the different thymic iNKT cell subsets have disparate but overlapping avidities for the PBS57–CD1d complex.

It is conceivable that these observed differences in avidities for the CD1d-foreign antigen PBS57 could also reflect differential avidities for the CD1d-self antigen(s) recognized during positive selection of the different iNKT cell subsets. Consequently, the strength of TCR signaling received by the different iNKT cell subsets during positive selection might be different as well. Surface expression of CD5 and CD6 molecules directly parallels the signaling intensity of the positively selecting TCR–MHC–ligand interaction^22–26^, while expression of Ly6C by CD4 T cells is inversely correlated with self-reactivity of their TCRs^27^. As seen in Fig. 1c, iNKT2 cells express the highest level of CD5 and CD6 proteins while they are negative for Ly6C. iNKT17 cells have lower levels of CD5 and CD6 expression than iNKT2 cells and are also Ly6C negative. Finally, iNKT1 cells express the lowest levels of CD5 and CD6 proteins of the three subsets, while >60% of the cells are also Ly6C positive. Thus, on the basis of expression of these 3 markers, it appears that the three iNKT cell subsets have received different strengths of signaling during selection in the thymus, with the following hierarchy iNKT2 > iNKT17 > iNKT1. We also examined Egr2 expression in the three iNKT cell subsets (Fig. 1c). The results show that iNKT2 cells express the highest level of Egr2 followed by iNKT17 and iNKT1 cells. These results are in agreement with the expression of GFP driven from the immediate early gene *Nr4a1* (Nur77) locus, which directly correlates with the strength of TCR stimulus^28^. Altogether, these results demonstrate that the different iNKT cell subsets receive signals of different strength during their development in the thymus and that the avidity of the TCR repertoires expressed by each subset positively correlates with this signaling strength.

### Development of iNKT subsets in mice with a fixed TCRβ chain

**Fig. 2:**
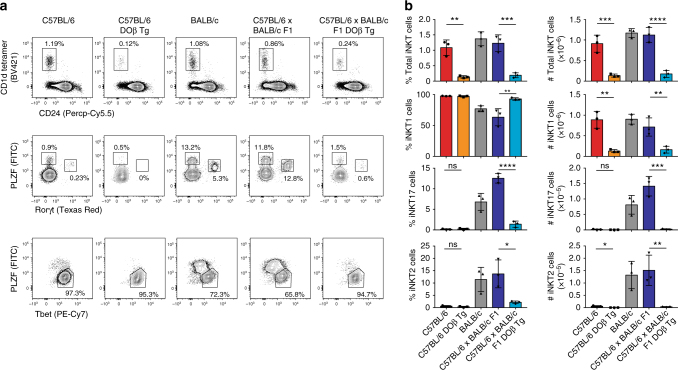
Development of iNKT cell subsets in DO11.10 TCRβ transgenic mice on the C57BL/6 or C57BL/6 × BALB/c F1 background. **a** The thymi of 8-week-old C57BL/6, C57BL/6 DO11.10 TCRβ transgenic, BALB/c, C57BL/6 × BALB/c F1, and BALB/c × C57BL/6 DO11.10 TCRβ transgenic mice were stained with anti-CD24 mAbs, PBS57–CD1d tetramers and mAbs specific for the transcription factors PLZF, Rorγt, and Tbet. Representative flow cytometry plots for the frequency of total mature iNKT cells and iNKT subsets in each genotype is shown. **b** Summary of the data shown in **a** with three mice per group. Data are mean ± SD. Significance was assessed using multiple comparison one-way analysis of variance (ANOVA) *p < 0.05; **p < 0.01; ***p < 0.001; ****p < 0.0001; ns, not significant. For clarity purposes, the only statistics shown are between mice with or without expression of the DO11.10 TCR transgene for a given genetic background

We examined whether the development of iNKT cell subsets would be disturbed if the specificity of the repertoire was fixed. We used transgenic mice that express the TCRβ chain derived from the OVA-IA^d^-specific clone DO-11.10 (DO). The composition of iNKT cell subsets in the thymus of 8-week-old B6 and BALB/c mice is different with BALB/c mice having large populations of iNKT2 and iNKT17 cells compared to B6 mice (Fig. 2)^2^. Interestingly, the B6 × BALB/c F1 strain recapitulates the BALB/c phenotype (Fig. 2), allowing us to analyze the effect of TCRβ DO expression on iNKT cell subset development in B6 mice and F1 (B6 × BALB/c) mice. Enforced expression of a single TCRβ chain strongly affects the development of iNKT cell subsets on both genetic backgrounds (Fig. 2). The proportions of iNKT2 and iNKT17 cells in DO11.10 TCRβ transgenic mice are largely reduced compared to those of control mice with polyclonal repertoires, demonstrating that the specificity of the iNKT TCR has a key role in the development of iNKT cell subsets.

### Vβ repertoire and Egr2 levels in thymic iNKT cell subsets

**Fig. 3:**
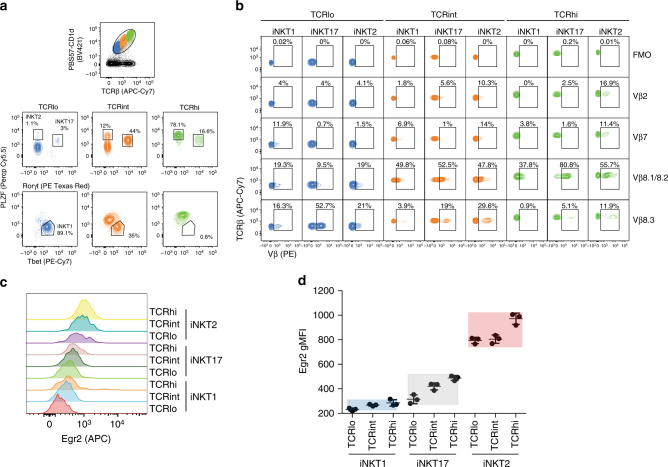
Thymic iNKT subsets with different TCR levels express different proportions of Vβ usage but discrete Egr2 levels. Cells from the thymus of 8-week-old BALB/c mice were stained with anti-TCRβ mAbs, PBS57–CD1d tetramers, mAbs specific for the transcription factors PLZF, Rorγt, and Tbet and the indicated anti-Vβ mAbs. **a** iNKT cells were subdivided based on expression of different TCR levels. The proportion of iNKT cell subsets for each level of TCR expression (TCR^low^ (blue), TCR^int^ (orange) and TCR^high^ (green)) is shown. **b** The percentage of Vβ usage found in each iNKT subset expressing three distinct TCR levels is shown with the fluorescence minus one control (FMO). Data are representative of three independent mice analyzed. **c** Example of Egr2 expression in each iNKT cell subsets expressing different levels of TCR. **d** The gMFI of Egr2 expression was recorded from three independent staining of iNKT cell subsets expressing different TCR levels from one individual mouse as gated in **a**. Three independent mice were analyzed in this way with similar results

In addition to its surface expression level, the nature of the TCR itself can affect the duration and the strength of TCR signaling^29^. Therefore, we assessed the percentage of Vβ usage for the three iNKT cell subsets expressing different avidities for the PBS57–CD1d tetramer. BALB/c thymic iNKT cells were electronically gated based on three discrete TCR levels (Fig. 3a). Although the correlation between avidity and iNKT cell subsets held generally true (Fig. 1a), each iNKT subset could nevertheless be found, albeit in very low frequencies in some instances, for each level of TCR expression (Fig. 3a). We examined the expression of Vβ2, Vβ7, Vβ8.1/8.2, and Vβ8.3 by the three iNKT cell subsets found for each level of TCR (Fig. 3b). In agreement with previous results^2^, we found that the proportion of Vβ usage is different between iNKT cells subsets. Unexpectedly, however, the proportion of Vβ usage within a given subset also varies between cells having different avidities for the PBS57–CD1d complex. For example, 52% of iNKT17 cells expressing low TCR levels use the gene segment Vβ8.3, while only 5.1% of iNKT17 cells that are TCR^hi^ are Vβ8.3^+^ (Fig. 3b). Likewise, 19% of TCR^lo^ iNKT2 cells use Vβ8.1/8.2 but this proportion increased to 55% for iNKT2 cells expressing high levels of TCRs (Fig. 3b). These results demonstrate that the repertoire of each iNKT cell subset is not only unique to a given subset but also varies as a function of the level of TCR surface expression. We also analyzed the levels of Egr2 expression in each iNKT subset expressing different TCR levels. Each iNKT cell subset expresses a limited range of Egr2 protein (Fig. 3c, d) that appears delimited by the levels of TCR surface expression for a given subset. For example, the few iNKT1 cells that are TCR^hi^ express Egr2 levels that remain lower than TCR^lo^ iNKT2 cells (Fig. 3d). Thus, it appears that each iNKT cell subset expresses a unique TCRβ repertoire modulated by the overall level of TCR expression on the cells but that the combination results in discrete signal strength.

### Thymic iNKT cell subsets in Zap70-mutant mice

**Fig. 4:**
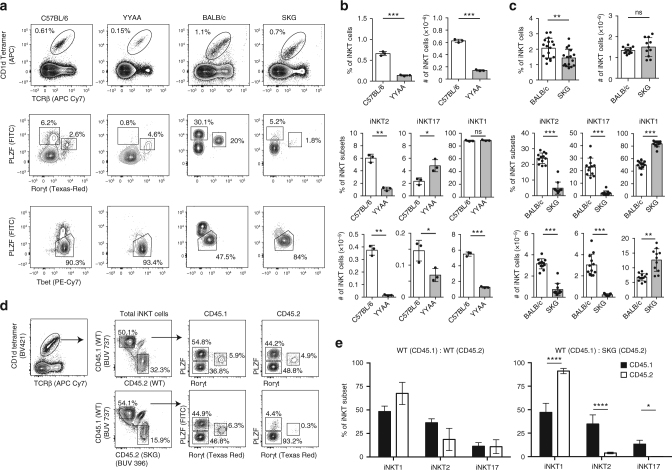
Cell-intrinsic deficiency in iNKT2 and iNKT17 subsets development in mice with Zap70 hypomorphic mutations. **a** Representative flow cytometry plots showing the frequency of total iNKT cells and iNKT cell subsets in the thymi of 8-week-old C57BL/6, C57BL/6 YYAA, BALB/c, and BALB/c SKG mice. Each genotype was analyzed independently and compared to wild-type control mice of the appropriate background. **b**, **c** Summary of the data shown in **a** with percentages and cell numbers of total iNKT cells and iNKT cell subsets in the various genotypes analyzed. Data are mean ± SD. Significance was assessed using the unpaired *t*-test. **d** Analysis of thymic iNKT cell subsets in competitive bone marrow chimera. Congenically marked bone marrow cells from BALB/c wild-type mice (CD45.1) were mixed in a 1:1 ratio with (CD45.2) BALB/c or SKG bone marrow cells and injected into lethally irradiated F1 (CD45.2 × CD45.1) BALB/c mice. The thymi of the chimera were analyzed for iNKT cell subset composition 8–10 weeks post reconstitution and representative flow cytometry plots are shown. After gating on total iNKT cells, the proportion of iNKT cell subsets derived from either CD45.2^+^ or CD45.1^+^ bone marrow cells is shown. **e** Summary of the data shown in **d** with ≥5 mice per group. Data are represented as mean ± SD. Significance was assessed using one-way analysis of variance (ANOVA) *p < 0.05; **p < 0.01; ***p < 0.001; ****p < 0.0001; ns, not significant

To further test the idea that strength of TCR signaling affects iNKT cell fate choice, we examined iNKT cell development in mouse models with hypo-responsiveness to TCR stimulation, the YYAA and SKG mice^30,31^. Both mutations diminish TCR signal strength and thus provide opportunities to analyze the effects of signal strength on iNKT cell differentiation on both B6 and BALB/c genetic backgrounds. Both strains show skewed development of iNKT subsets (Fig. 4). In the thymus of YYAA mice, iNKT2 cells are largely decreased proportionally and numerically compared to the B6 controls (Fig. 4a, b), while in SKG mice, which have a more profound impairment of TCR signaling than the YYAA mice^30^, most of the iNKT cells are iNKT1 cells in striking contrast to those in age-matched control BALB/c mice (Fig. 4a, c). Because differences in subset ratios are stronger between BALB/c and SKG mice, subsequent experiments are only carried out with these two strains.

### Cell-intrinsic Zap70 affects iNKT subset development

To determine whether the SKG mutation effects on iNKT cell subset development are cell intrinsic or extrinsic, we generated competitive bone marrow chimeras. BALB/c-derived bone marrow cells produce all three iNKT cell subsets with large proportions of iNKT2 (40–50%) and iNKT17 (5–6%) cells (Fig. 4d). By contrast, SKG-derived iNKT cells are composed mostly of iNKT1 cells, with poor representation of iNKT2 (4%) and iNKT17 (0.3%) cells. These results demonstrate that iNKT cell precursors need to receive adequate TCR signaling mediated through Zap70 in order to develop efficiently into the iNKT2 and iNKT17 lineages and that cell extrinsic signals in the thymus cannot compensate for this deficiency.

### Zap70 activity-dependent gene expression in stage 0 cells

**Fig. 5:**
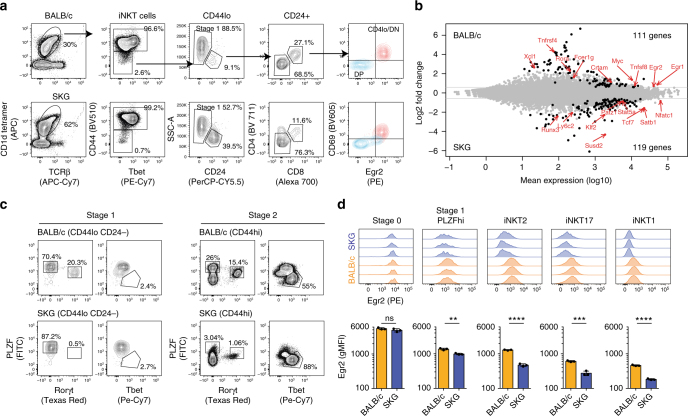
Differential gene expression in iNKT cells of BALB/c and SKG mice. **a** Gating strategy used to identify and sort stage 0 iNKT cells in BALB/c (top) and SKG (bottom) mice. Enrichment of iNKT cells from the thymi of BALB/c or SKG mice was achieved after staining with APC-labeled PBS57–CD1d tetramer and enrichment using anti-APC magnetic beads. Stage 0 iNKT cells were defined as TCRβ^+^ PBS57–CD1d tetramer^+^ CD44^−^ CD24^+^ CD4^lo^ CD8^−^ CD69^+^. As seen on the figure, these cells are also Egr2^+^ (red) in contrast with the CD4^+^CD8^+^ (blue) population that invariably contaminates the cell preparation. **b** Mean average plot of genes expressed differentially in stage 0 iNKT cells isolated from SKG mice relative to their expression in such cells from BALB/c control mice. Data are representative of one experiment with three biological replicates per genotype. 238 genes (displayed in black) were found to be differentially expressed >1.5-fold with a false discovery rate <0.1. **c** Analysis of transcription factor (PLZF, Rorγt, and Tbet) expression in stage 1 (CD44^−^ CD24^−^) and stage 2 (CD44^+^) iNKT cells from the thymi of BALB/c and SKG mice. Data are representative of >5 mice analyzed for each genotype. **d** Analysis of Egr2 expression level in BALB/c (orange) and SKG (purple) iNKT cells at different developmental stages. Results from three mice of each genotypes analyzed independently are shown. The mean ± SD of Egr2 gMFI in each iNKT cell developmental stage in BALB/c and SKG mice is shown. Significance was assessed using the unpaired *t*-test. **p < 0.01; ***p < 0.001; ****p < 0.0001; ns, not significant

To determine whether changes in strength of TCR signaling might have affected the developmental program of iNKT cells, we used RNA-Seq to compare gene expression in the earliest iNKT cell progenitors, just after positive selection (stage 0 cells), between BALB/c and SKG mice. iNKT cells were enriched using PBS57–CD1d tetramer and magnetic beads (Fig. 5a). Stage 0 iNKT cells were defined as CD44^lo^, CD24^+^, CD4^low/−^, CD8^−^, CD69^+^, and sorted using these markers. This sorting strategy eliminates DP thymocytes that invariably contaminate the CD24^+^ fraction and that do not represent genuine stage 0 iNKT cells that are actively signaling (Fig. 5a and Supplementary Fig 2). Confirmation that these cells are genuine iNKT precursors is provided by the analysis of the sequencing reads that are mapped to the TCRα locus (Supplementary Fig 3). We detected statistically significant differential expression (FDR < 0.1) of 238 genes between the two genotypes (Fig. 5b), with a significant enrichment for genes implicated in cell activation and immune cell development (Supplementary Fig 4). Amongst the differentially regulated genes, several are known regulators of iNKT cell development, including *Egr1* and *Egr2*, *Myc*, *Tcf7*, *Klf2*, *Runx3*, and *RORa*. Interestingly, several factors involved in chromatin remodeling and transcriptional regulation, which are induced during positive selection of T lymphocytes (*Patz1* and *Satb1*), are also differentially regulated. Altogether, these results demonstrate that TCR hypo-responsiveness affects iNKT cell development even at the earliest stage of commitment.

### Reduced Egr2 expression in iNKT cells from SKG mice

In agreement with the RNA-seq data, the levels of Egr2 in stage 0 iNKT cells of SKG mice (gMFI 4866 ± 421) are slightly reduced (10%) compared to those in BALB/c animals (gMFI 5432 ± 293), although the results do not reach statistical significance (Fig. 5d). According to the conventional “staging” system of iNKT cell development^3^, immediately following stage 0, precursor cells (iNKTp) are CD44^lo^ CD24^−^ CD69^−^ and PLZF^hi^ ^2^. The cells subsequently reach stage 2 (CD44^hi^), which contains functionally mature iNKT cell subsets^3^. We observed that the differences in the levels of Egr2 expression between BALB/c and SKG mice are more pronounced as the cells progress in their maturation (Fig. 5c, d). iNKTp cells from SKG mice express 1/3 less Egr2 than their counterpart cells in wild-type mice (Fig. 5d). While iNKT2 cells express the highest levels of Egr2 of the three iNKT subsets in BALB/c mice (Figs. 1 and 5d), its level of expression in the few iNKT2 cells of SKG mice (gMFI 469 ± 35) is comparable to the levels found in BALB/c iNKT1 cells (gMFI 459 ± 15). Expression of PLZF and Rorγt in iNKT2 and iNKT17 cells respectively, are also lower in SKG mice (Fig. 5d). Altogether, these results demonstrate that during development of iNKT cells in SKG mice, Egr2 is induced at near normal levels in stage 0 iNKT cells but its expression is not sustained effectively as the cells mature.

### Modulation of Egr2 and PLZF induction in vitro

**Fig. 6:**
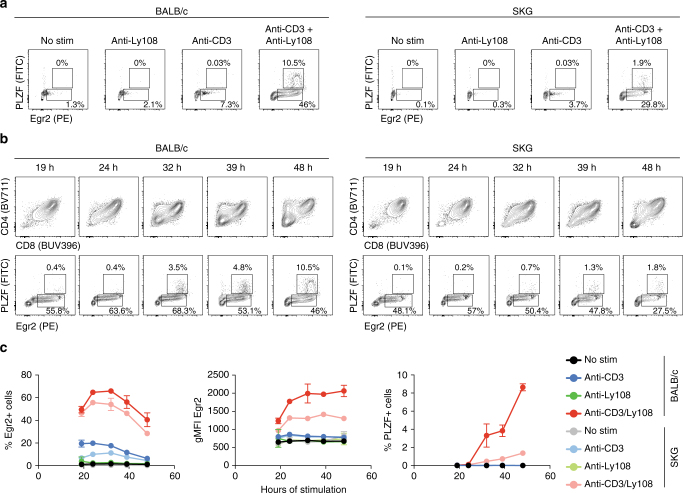
Induction of Egr2 and PLZF in pre-selection DP thymocytes stimulated with plate-bound anti-CD3 and anti-Ly108 mAbs is affected by the SKG mutation. **a** Expression of Egr2 and PLZF proteins in pre-selection BALB/c or SKG DP thymocytes stimulated with media alone (no stim), plate-bound anti-CD3, anti-Ly108, or anti-CD3 + anti-Ly108 for 48 h. At the end of the stimulation, cells were stained using anti-CD4, -CD8, -Egr2, -PLZF mAbs, and a viability dye. The percentages of live cells that are Egr2^+^ and PLZF^+^ are indicated for each stimulatory condition. **b** Time course of CD4, CD8, Egr2, and PLZF expression in pre-selection BALB/c or SKG DP thymocytes stimulated with plate-bound anti-CD3 + anti-Ly108 mAbs. Data are shown for live cells in each conditions. **c** Summary of the data shown in **b** for each stimulatory condition. The percentage of Egr2^+^ cells, the gMFI of Egr2 expression, and the percentage of PLZF^+^ cells are shown. Each experimental condition was tested in duplicate wells and the data are representative of greater than three independent experiments with similar results

While induction of Egr2 expression in developing T cells is known to occur downstream of TCR signaling, whether PLZF is induced in an Egr2-dependent manner in response to TCR signaling has been controversial^15,32^. Pre-selection DP thymocytes from the thymus of BALB/c and SKG mice were stimulated with anti-CD3, anti-Ly108 or a combination of both, and followed over time for the expression of Egr2 and PLZF. As seen in Fig. 6, TCR stimulation alone of BALB/c DP thymocytes triggers a few cells to produce Egr2 but its level of expression, which peaks around 40 h post stimulation, remains low. In these conditions, we do not detect PLZF expression at any time point post stimulation. As reported previously^17,18^, co-stimulation with the SLAM family member Ly108 increases the proportion of cells expressing Egr2 as well as the level of expression per cell. Interestingly, cells expressing the highest levels of Egr2 begin to express the PLZF protein around 32 h post stimulation with the proportion of PLZF^+^ cells increasing as a function of time. These results establish that the transcription factor PLZF can be induced in DP thymocytes following TCR/Ly108 stimulation and that only the cells that sustain the highest levels of Egr2 can also express PLZF. Stimulation of SKG DP cells in the same conditions induces a slightly lower percentage of Egr2^+^ cells than the BALB/c cells (Fig. 6b, c) but the levels of Egr2 induced never reach the levels found in the BALB/c cells at any time point post stimulation, and few PLZF^+^ cells are detected in these conditions. These results recapitulate ex vivo observations (Fig. 5) and further support the hypothesis that modulation of TCR signal strength can affect iNKT cell subset differentiation, in part by modulating expression of Egr2 and PLZF.

### Zap70 activity affects chromatin landscape of iNKT subsets

**Fig. 7:**
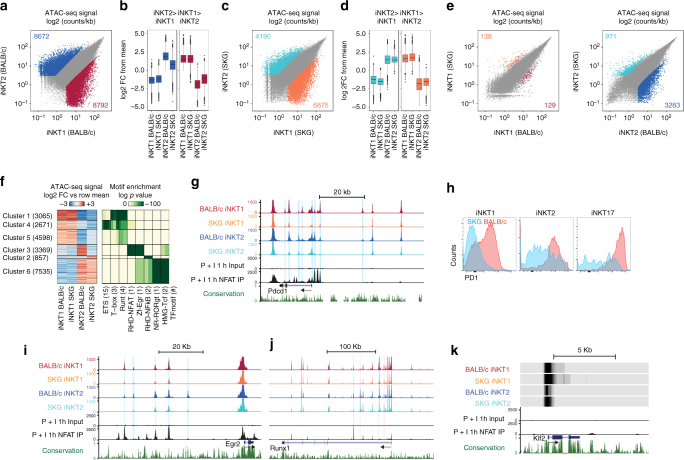
Genome-wide chromatin landscapes of iNKT1 and iNKT2 subsets are affected by the SKG mutation. **a**, **c** Scatterplots of mean ATAC-seq counts per peak comparing iNKT1 and iNKT2 subsets from BALB/c (**a**) or SKG (**c**) mice. **b**, **d** Boxplots of ATAC-seq counts per plots from the indicated samples (labeled at the bottom) at common or differentially accessible regions from the comparison labeled above. Box indicates interquartile range with whiskers ± 1.5 times this range and outlier points. **e** Scatterplots of mean ATAC-seq counts per peak comparing iNKT1 from BALB/c vs SKG mice and iNKT2 subsets from BALB/c vs SKG mice. **f** k-means clustered log2 fold-change from mean ATAC-seq signal for all differentially accessible regions compared to all differentially accessible regions ini NKT1 and iNKT2 cells from BALB/c and SKG mice. The enrichment of known motifs within each cluster of differentially accessible regions compared to all differentially accessible regions in iNKT1 and iNKT2 cells from BALB/c and SKG mice. All motifs with an enrichment log *p*-value <−35 and found in 10% or more regions in at least one cluster are shown. **g** Mean ATAC-seq coverage at the *Pdcd1* locus with a scale 0–1000 for ATAC-Seq tracks. NFAT ChIP-seq coverage from CD8 T cells stimulated or not with PMA + ionomycin for 1 h^37^ are also shown. **h** Flow cytometry analysis of PD1 expression on thymic iNKT cell subsets from BALB/c (red) and SKG (blue) mice. **i** Mean ATAC-seq coverage at the *Egr2* locus with a scale 0–1000 for ATAC-Seq tracks. **j** Mean ATAC-seq coverage at the *Runx1* locus with a scale 0–1000 for ATAC-Seq tracks. For **g**, **i**, and **j**, ATAC peaks with differential signals where BALB/c > SKG mice are highlighted in blue, while peaks with signals SKG > BALB/c mice in at least one subset are in red. **k** ATAC-Seq of chromatin accessibility at the transcription start site of *Klf2* in thymic iNKT1 and iNKT2 subsets from BALB/c and SKG mice

To assess how TCR signal strength might influence lineage differentiation via regulatory elements, we compared chromatin accessibility between BALB/c and SKG iNKT1 and iNKT2 cells with ATAC sequencing (ATAC-seq). iNKT1 and iNKT2 cell subsets show a unique chromatin landscape, with regions of accessibility specific to each subset. In agreement with their distinct functionalities, the accessibility of subset signature genes (*Ifnγ*, *Tbx21*, *Cxcr3*, *Ccr5*, *Il2rb*, *Fasl*, *Bcl2*, *Il2*, *Zbtb16*, *Slamf6*, *Il17rb*, *Il6ra*, *Ccr7*) is different between subsets (Supplementary Figs. 5 and 6). For example, the chromatin landscapes of the *Ifnγ* and *Tbx21* genes, encompassing not only their promoters but also intragenic and intergenic regions extending as far as several kilobases away, are clearly accessible in iNKT1 but not in iNKT2 cells. By contrast, the chromatin landscape surrounding the *Zbtb16* (encoding PLZF), *Slamf6* (encoding Ly108), *Il17rb*, *Il6ra*, and *Ccr7* genes is clearly more accessible in iNKT2 than iNKT1 cells, in agreement with the differential expression of these genes between these two iNKT subsets^33–35^. Globally, 17,464 regions (out of a total of 90,051 accessible regulatory regions identified) are differentially accessible between BALB/c iNKT1 and iNKT2 cells (Fig. 7a). Similarly, 11,065 regions are differentially accessible between SKG iNKT1 and iNKT2 cells (Fig. 7c). Regions that are more accessible in BALB/c iNKT1 than in BALB/c iNKT2 cells also tend to be more accessible in SKG iNKT1 than in SKG iNKT2 cells (Fig. 7b, d). Conversely, the same holds true for regions that are more accessible in iNKT2 over iNKT1 cells (Fig. 7b, d). These results suggest that the mechanisms involved in setting up the chromatin accessibility landscape specific to each iNKT cell subset are conserved between BALB/c and SKG mice. To ask whether there are specific changes in chromatin accessibility in iNKT cells from SKG mice, we compared the ATAC-seq data between BALB/c and SKG backgrounds for each subsets. In iNKT1 cells, we identified 267 regions that are differentially accessible between BALB/c and SKG, suggesting that reduced TCR signaling only minimally impacts the differentiation of the iNKT1 lineage (Fig. 7e). By comparison, 4254 regions are differentially accessible between BALB/c and SKG iNKT2 cells (Fig. 7e), with >75% of these regions only accessible in BALB/c cells with reduced accessibility in SKGs. To group regulatory elements with similar profiles on their relative signal in each subsets between BALB/c and SKG mice, we partitioned all differentially accessible regions into 6 clusters using k-means. Regions in clusters 1, 4, and 5 define the iNKT1 chromatin landscape signature and are significantly enriched for T-box, Runt, and ETS binding sequences, while clusters 6 and 3 are associated with iNKT2 cells and are enriched for Tcf, Rorγt, NFκB, Egr and NFAT-binding sites (Fig. 7f). Interestingly, cluster 3, which defines peaks found in BALB/c iNKT2 cells but absent from SKG iNKT2 cells, is strongly enriched for NFAT and Egr binding sites (Fig. 7b), suggesting that this unique signature is affected by the SKG mutation (Fig. 7b). Conversely, cluster 2, which defines peaks found in SKG iNKT cells but absent from BALB/c iNKT cells, shows an enrichment for NFκB-binding sites (Fig. 7f). These results suggest that in addition to losing the NFAT/Egr signature, potentially due to a quantitative decrease in TCR signaling, the SKG mutation also alters the signal received by the cells in a qualitative manner.

PD1 expression in activated CD8 T cells is controlled by regulatory regions in the *Pdcd1* locus. Accessibility to these regions has been directly linked to the binding of NFAT^36,37^. The same regulatory regions are found accessible in BALB/c iNKT cells (Fig. 7g), with small differences between BALB/c iNKT1 and iNKT2 cells, suggesting that the locus might be differentially regulated in these subsets (Fig. 7g). Importantly however, accessibility to these sites is largely diminished in both iNKT subsets derived from SKG mice (Fig. 7g). Consequently, PD1 is poorly expressed on SKG iNKT cells compared to the wild-type cells (Fig. 7h). These results illustrate the loss of the NFAT signature in iNKT cells from the SKG mice (Fig. 7f).

While the chromatin accessibility profile of the *Zbtb16* locus (encoding PLZF) is not disturbed by the SKG mutation in any iNKT subset (Supplementary Fig 6), we noticed that the loci surrounding two of the transcription factors known to regulate the expression of PLZF in iNKT cells (Egr2 and Runx1^15,38^) are affected (Fig. 7i, j). Within 50 Kb of the *Egr2* transcription start, we identified five putative regulatory regions, some of which are shown to directly bind NFAT in CD8 T cells (Fig. 7i). Importantly, accessibility to these five regions is different between BALB/c iNKT1 and iNKT2 cells, suggesting that control of Egr2 expression between iNKT cell subsets involves different regulatory mechanisms. Nevertheless, for both iNKT subsets, the SKG mutation affects the pattern of chromatin accessibility of putative *Egr2* regulatory regions (Fig. 7i). These results are consistent with the diminished levels of Egr2 expression within these cells (Fig. 5d). Similar results are found for the *Runx1* locus, where subset-specific chromatin accessibility peaks can clearly be delineated (Fig. 7j). Two of these regulatory regions (one of them directly binds NFAT in CD8 T cells) are affected by the SKG mutation (Fig. 7j), suggesting that like Egr2, Runx1 expression might also have been disturbed by the SKG mutation. Finally, analysis of the transcription starting site (TSS) of the *Klf2* gene demonstrates more accessibility in BALB/c iNKT1 over iNKT2 cells, in agreement with the known differential expression of this gene between iNKT1 and iNKT2 cells^2,35^. Accessibility at the TSS is largely increased in both iNKT subsets derived from the SKG mice (Fig. 7k). Such increase in Klf2 expression is already apparent in stage 0 SKG iNKT cells (Fig. 5b).

To identify potential gene targets of the regulatory elements that are differentially accessible between BALB/c and SKG iNKT cells, we performed gene ontology analysis using GREAT (Genomic Regions Enrichment of Annotation Tool)^39^ (Supplementary Fig 7). For cluster 3, we observe a significant enrichment of gene onthology (GO) terms for the regulation of kinase activity and Notch signaling (Supplementary Fig 7 and Supplementary Table I). Several genes encoding proteins involved in the regulation of mitogen-activated protein kinases (MAPK) activity, including the dual-specificity protein phosphatases Dusp4, Dusp5, and Dusp6^40^, as well as the MAPK/Erk pathway regulators sprouty proteins 1 and 2^41^ show differential chromatin accessibility profiles between iNKT cell subsets that are affected by the SKG mutation (Supplementary Fig. 8). Similar observations are made for the Notch pathway-associated genes, *Hey1*, *Jag1*, *Ovol2*, and *Rbpj* (Supplementary Fig 9). Interestingly, several of these peaks (but not all) bind NFAT in CD8 T cells (Supplementary Figs. 8 and 9). Altogether, these results suggest a potential link between the reduced TCR signaling in SKG mice and alterations of MAPK/Erk activity and Notch signaling in having a role in the development of iNKT cell subsets.

## Discussion

In the thymus, T cell precursors are selected to undergo different fates and functions such as becoming γδ or αβ T cells^42^, CD4 or CD8 T cells^43^, positively selected or clonally deleted^44^, and “conventional” or “agonist-selected” lymphocytes^45^. In these processes, TCR signals cooperate with co-stimulatory molecules, cytokines, chemokines, integrins, and metabolic signals to regulate the generation of T cells with very different phenotypes and functions.

Here, we demonstrate that TCR signal strength is an essential variable in shaping the development of iNKT cell subsets. The three major thymic iNKT subsets generally have different avidities (a combination of TCR expression levels and TCR affinities) for the PBS57–CD1d complex, which is correlated with different levels of proteins whose surface expression levels reflect the TCR signal strength initially perceived during selection^22,26–28^. Thus, as reported previously for conventional T cells^23,25^, the strength of self-reactivity appears directly related to the strength of TCR binding to the α-galactosylceramide analog (PBS57) presented by CD1d. Interestingly, αGC was recently identified in the mouse thymus where it was proposed to have a role in the positive selection of iNKT cells^46^. iNKT2 cells stain the brightest with the PBS57–CD1d tetramer, followed by iNKT17 cells and finally iNKT1 cells. While the quantity of surface TCR available for ligand interaction reflects this differential staining, it is possible that it also mirrors the signaling capacity of the iNKT cell subsets, with high TCR expressing cells capable of sustaining TCR engagement for longer period of time that in turn affect the kinase activity of Zap70^29^. A more direct measure of TCR signal strength received by each subset, through direct staining of the TCR-induced transcription factor Egr2, further reveals that iNKT2 cells indeed receive stronger and/or more persistent TCR signaling than iNKT17 and iNKT1 cells in vivo. This differential Egr2 expression appears to be regulated at the transcriptional level^33^.

As reported previously^2^, the frequency of Vβ usage varies among iNKT cell subsets. Interestingly, Vβ usage within a given subset varies as a function of TCR level as well. Notwithstanding these variations, discrete levels of Egr2 expression are observed in each iNKT cell subset, irrespective of TCR expression levels. These results support the idea that the actual Vβ-Vα usage (and perhaps CDR3β sequence) expressed by each iNKT cell precursor is responsible for the differential signaling observed, in good agreement with recent findings showing that iNKT cell subset development is affected by the biophysical properties of the TCR–antigen–CD1d interaction^47,48^. That said, we do not observe disparate TCR expression levels on the surface of stage 0 precursors. It is possible that small differences in TCR expression levels on iNKT cell precursor cells exist, but cannot be resolved with the current sensitivity of flow cytometry. Additionally, it is also possible that, for equivalent levels of TCR expression, the exact nature of each TCR dictates the signal strength received by each precursor and that as a consequence of this signaling, each precursor cell modulates its TCR surface level as the cell progresses through development^49^. In support of this hypothesis, iNKT2 cells express the highest levels of TCR and also express the highest levels of GATA3^50^. While GATA3 is an autoactivator of its own expression^51^, it also directly binds to several gene loci, including *Tcra*, *Tcrb*, *Cd3d*, and *Cd3g*^52^, and controls surface TCR expression level^52,53^. This might provide a positive regulatory loop whereby cells that signal strongly would initially induce high levels of GATA3, leading to increased surface expression of the TCRs and increased avidity for their cognate ligands.

To test whether differential TCR signaling is required for the proper development of iNKT cell subsets, we either reduced the TCR repertoire diversity of iNKT cells through the use of TCRβ transgenic mice or we reduced TCR signaling capacity of the polyclonal repertoire through the use of mice containing hypomorph mutations in the proximal tyrosine kinase Zap70. In both cases, we observe that limiting the strength of TCR signaling or the diversity of the TCR repertoire, preferentially affects, in a cell-intrinsic and genetic background independent manner, the development of iNKT2 and iNKT17 cells, while iNKT1 cells are less disturbed. These results are in agreement with the idea that the stronger TCR signaling observed in the two former subsets is indispensable to their proper development and cannot be replaced by external cues. Transcriptomic analysis of the stage 0 iNKT population from wild-type and Zap70-mutant mice demonstrates changes in expression of several transcripts coding for genes that have previously been involved in iNKT cells development, including *Egr*, *Myc*, *Tcf7,* and *Klf2*. For example, stage 0 iNKT cells from SKG mice are enriched 4.5-fold (*p*_adj_ < 0.07) in *Klf2*-encoding transcripts compared to their wild-type counterpart. Interestingly, *Klf2* deficiency results in an increase in the proportion of iNKT2 cells^2^. Conversely, Myc-encoding mRNA is increased in cells derived from the BALB/c mice compared to SKG mice (2.1-fold, *p*_adj_ < 10^−6^). In the absence of Myc, development of iNKT1 cells is blocked and the remaining thymic iNKT cells have a phenotype reminiscent of iNKT2 cells instead^54^, suggesting a role for Myc in the subset differentiation of iNKT cells. These experiments argue that reduced TCR signal strength during the positive selection of iNKT cells changes the expression levels of several genes that affect subsequent subset differentiation.

Surprisingly, we observe only small reductions in expression of *Egr2* (1.6 fold, *p*_adj_ < 0.5) and *Egr1* mRNA (1.8 fold, *p*_adj_ < 0.0002) in stage 0 iNKT cells from SKG mice compared to the BALB/c mice. Analysis of Egr2 protein levels in stage 0 cells confirmes these results. This is unexpected because both genes are well known downstream targets of TCR signaling during thymic selection^55^ and have been previously implicated in iNKT cell development^14–16^. While the precise role of Egr1 remains unclear and appears subtle in wild-type iNKT cells^14,16^, lack of Egr2 expression by contrast leads to severe deficiency early in iNKT cell development^15^. Nevertheless, reduction of TCR signaling only minimally affects the induction of Egr2 in early iNKT progenitors. However, analysis of the subsequent stages of development expose a severe loss of Egr2 expression in iNKT cell from SKG mice compared to the levels observed in the same cells from BALB/c mice. Thus, in the presence of reduced Zap70 activity, the acute induction of Egr2 appears conserved but its prolonged expression is not. This phenomenon can be mimicked in vitro upon co-stimulation of pre-selection DP thymocytes with anti-Ly108 and anti-CD3 mAbs. While very high levels of Egr2 expression can be achieved acutely in these conditions, more than 24 h of sustained stimulation is required for the highest Egr2-expressing cells to express PLZF. Stimulation of SKG cells in the same conditions does not sustain the levels of Egr2 expression over time and few SKG DP thymocytes induce PLZF. Several conclusions can be reached from these experiments. First, expression of the PLZF protein can be induced in pre-selection DP thymocytes in vitro and requires prolonged co-signaling through the TCR and Ly108 molecules. These results support the hypothesis that TCR signaling can instruct the development of “innate” (as defined by PLZF expression) lymphocytes^15,56^, including iNKT cells^3^, in contrast with other data^32^. Second, only the highest Egr2-expressing cells are able to express the PLZF protein, expanding upon previous results where gene expression was examined by qPCR in entire cell populations^17^. Third, high Egr2 expression levels are necessary but not sufficient to induces PLZF, as it needs to be sustained for more than 24 h. We propose that reduced TCR signaling in SKG mice affects the longevity of Egr2 expression potentially through epigenetic mechanisms involving downstream TCR signaling molecules. Because the persistence of TCR signaling sustains cross talk with other pathways that can further modulate T cell fate ^57–60^, other events capable of modulating the strength and/or duration of signaling in iNKT cell precursors could also affect subset specification.

Global epigenetic information is more stable than gene expression profiles and propagates information over time during development and differentiation^61^. Changes in chromatin accessibility between iNKT1 and iNKT2 cells suggest lineage-specific activity of regulatory elements. Several regions surrounding iNKT1-expressed genes are only accessible in iNKT1 but not in iNKT2 cells. Globally, these iNKT1-specific chromatin accessibility regions are enriched for Runt, ETS and T-box binding motifs, suggesting that transcription factors belonging to these families might contribute to iNKT1 development by binding to these differentially accessible regions. Importantly, few differences in chromatin accessibility are observed between iNKT1 cells of BALB/c or SKG mice, implying that reduced TCR signaling does not appreciably affect the chromatin accessibility necessary to establish the iNKT1 program. Conversely, we detected accessible regulatory elements that are specific to iNKT2 cells. Generally, iNKT2-specific regions are enriched for Tcf, Rorγt, Egr, and NFAT-binding sites, indicating that commitment to the iNKT2 program might instead require binding of these transcription factors to these regions. Remarkably, part of this iNKT2 signature is affected by the SKG mutation with 30% of BALB/c iNKT2-specific regions being inaccessible in SKG iNKT2 cells. These regions are enriched for Egr and NFAT-binding sites, two transcription factors with activity directly linked to TCR signaling. These results suggest that efficient development of iNKT2 cells requires higher activity of these two transcription factors for binding to these regions. Altogether, our data point towards a model of iNKT cell subset development where variable TCR signaling might induce epigenetic changes at NFAT and Egr binding sites that may have corresponding effects on gene expression and differentiation. Whether these effects are due to direct binding of these transcription factors and/or binding in collaboration with other transcription factors at these sites remains to be determined.

Interestingly, the chromatin accessibility surrounding the *Egr2* locus (and *Runx1*) is different between iNKT cell subsets, with potential regulatory enhancer regions only accessible within specific subsets. Furthermore, accessibility at several of these enhancer regions is diminished and/or absent in SKG-derived cells. Thus, modulation of accessibility to these enhancer regions might have affected gene expression between subsets and between BALB/c and SKG mice. Specific deletion of these enhancer regions in vivo will be necessary to formally test this hypothesis. However, because transcriptional regulation of PLZF expression in iNKT cells has been linked to Egr2^15^ and Runx1^38^, reduced expression of these two transcription factors in SKG cells would be expected to affect the expression of PLZF and thereby development of iNKT2/17 cells as well.

Our ATAC-seq analysis of iNKT2 cells in BALB/c and SKG mice also reveals that expression of several genes coding for regulators of the MAPK/Erk pathway might be affected by the SKG mutation. These might be implicated in feedback circuitry mechanisms essential in modulating the persistence of the signal^62^, as Egr2 induction downstream of TCR also requires the Ras/MAPK/Erk pathway^14^. A stronger resurgence of Erk phosphorylation is observed in the stimulation of pre-selection DP thymocytes in the presence of Ly108 co-stimulation^17^, suggesting a potential role in regulating Erk oscillations over time and as a consequence, possibly, the dynamics of epigenetic changes and gene transcription in the cells^63^.

Finally, the Notch signaling pathway has an important role in the differentiation of T cell effector subsets^64^. The chromatin accessibility surrounding several genes involved in this pathway is also affected by the reduced TCR signaling. While it is currently unclear whether any of these potential enhancer regions modulate gene activity, the results nevertheless suggest a potential cross talk between TCR and Notch in further modulating signaling and the differentiation of iNKT subsets in the thymus^65^. Future experiments will explore these possibilities.

## Methods

### Mice

The SKG mice on the BALB/c background^31^, and YYAA mice on the C57BL/6 background^30^ have been described previously. IL-4 reporter mice KN2^66^ backcrossed to the BALB/c background and the YYAA mice were graciously provided by Dr. Lee Reinhardt and Dr. Arthur Weiss, respectively. TCR transgenic mice on the C57BL/6 background expressing the TCRβ chain from the DO11.10 hybridoma have been described previously^67^. These mice were crossed with BALB/c mice to generate F1 expressing the transgene or not. Wild-type F1 littermates were used as controls. C57BL/6, BALB/c, and CD45.1 congenic BALB/c mice (CByJ.SJL(B6)-Ptprc^a^/J) were purchased from Jackson Laboratories. All mice were used between 8 and 10 weeks and were age-matched for each experiment. All mice were raised in a specific pathogen-free environment at the Biology Resource Center in National Jewish Health and the Office of Laboratory Animal Research at UCD. All animal procedures were approved by the NJH (AS2780-10-16) and UCD (B-64314(05)1E) Institutional Animal Care and Use Committees and were carried out in accordance with the approved guidelines.

### Thymocyte isolation and flow cytometry

Single-cell suspensions were prepared from the thymus by manual disruption using syringe plunger. PBS57-CD1d1 tetramer was obtained from the National Institutes of Health Tetramer Core Facility. The complete list of surface antibodies used is as follows: Anti-CD5 (53-7.3), anti-CD6 (BX222), anti-Ly6c (AL-21), anti-Vβ2 (B20.6), anti-Vβ7 (TR310), anti-Vβ8.1-8.2 (MR5-2), anti-Vβ8.3 (1B3.3), anti-Egr2 (erongr2), anti-CD24 (M1/69), anti-CD69 (H1.2F3). From BD Biosciences: anti-TCRβ (H57-597), anti-CD8α (53-6.7); from BioLegend: anti-CD44 (IM7); anti-CD4 (RM4-5). Surface antibody staining was done then cells were fixed and permeabilized using the FoxP3 buffer set (eBioscience). Fixed and permeabilized cells were incubated with intracellular antibodies including anti-PLZF (Mags.21F7; eBioscience), anti-Tbet (4B10; BioLegend), and anti-Rorγt (Q31-378; BD Biosciences). Cells were analyzed on a BD LSRFortessa (BD Biosciences) and data were processed with FlowJo software (TreeStar).

### Enrichment of CD1d-reactive thymocytes

Thymocytes were enriched for PBS57–CD1d-reactive cells by incubating thymocyte cell suspensions with PE or APC conjugated PBS57–CD1d tetramers for 45 min at 4 °C, then incubated with anti-PE or anti-APC magnetic microbeads (Miltenyi Biotec) for 15 min at 4 °C, followed by separation by using an autoMACS Pro Separator (Miltenyi Biotec) according to manufacturer’s instructions. Cells were first stained for surface markers and then intracellular stained for flow cytometric analysis.

### Bone marrow chimeras

Bone marrow cells from wild-type congenic BALB/c mice (CD45.1), BALB/c mice (CD45.2), and SKG (CD45.2) mice were harvested and depleted of CD90-expressing cells using magnetic beads. Bone marrow cells from wild-type CD45.1 mice were mixed at 1:1 ratio with either cells derived from wild-type CD45.2 mice or SKG mice. Overall, 5 × 10^6^ cells were injected intravenously into lethally irradiated wild-type BALB/c (CD45.1 × CD45.2) recipient mice (1000 rads). Eight to ten weeks after reconstitution, chimeric mice were euthanized and thymocytes were analyzed by flow cytometry.

### In vitro stimulation of pre-selection DP thymocytes

Total thymocytes from BALB/c and SKG mice were incubated with PE-labeled anti-CD127 (A7R34), anti-CD3 (145-201), anti-CD25 (PC61.5), anti-CD69 (H1.2F3), and PBS57/CD1d tetramers for 20 min at room temperature, washed and depleted using anti-PE magnetic beads and autoMACS pro separator (Miltenyi Biotec). Overall, 5 × 10^5^ pre-selection DP thymocytes (purity > 95%, verified by flow cytometry analysis for each experiment) were then placed in wells of 96-well plates some of which had been coated overnight with 1 µg/ml anti-CD3 (2C11) ± 5 µg/ml anti-Ly108 (13G3-19D). At different time points post stimulation, cells were stained with surface markers CD4 and CD8, fixed with the Foxp3 fixation/permeabilization kit and stained intracellularly for Egr2 and PLZF. Each condition was tested in duplicate for each independent experiment.

### RNA-Seq

Stage 0 iNKT cells (PBS57–CD1d tetramer^+^, CD44^−^, CD24^+^, CD69^+^, CD4^lo/−^, CD8^−^) were sorted from the pooled thymi of three BALB/c or SKG mice (~1 × 10^3^ cells were recovered) directly into Trizol buffer. Three independents samples were generated for each genotype. RNA quality was evaluated with the Bioanalyzer RNA pico kit (Agilent Technologies). Libraries were prepared from polyA^+^ RNA using the Whole Transcriptome Amplification Sequencing Technology SEQR kit (Sigma-Aldrich). Briefly, purified RNA was reverse transcribed using SuperScript II, Oligo dT30 VN primers, and template switching primers. A preamplification step of eight PCR cycles was performed using the Kapa HiFi Hotstart kit (Kapa Biosystems). The PCR product was purified using AMPureXP beads (Beckman Coulter) and 1 ng was further used for library preparation using the Nextera XT LibraryPrep kit (Illumina). Tagmented DNA was amplified with a 12-cycle PCR and again purified with AMPureXP beads. Library size distribution and yield were evaluated using the Bioanalyzer high-sensitivity DNA kit. Libraries were pooled at equimolar ratios and sequenced with the rapid run protocol on a HiSeq. 2500 (Illumina) with 50-nt single end cycling.

### RNA-Seq analysis

Reads were aligned using Hisat2 (v.2.1.0) with unstranded default parameters and the primary GRCm38 genome assembly (downloaded 08/26/2016). Reads overlapping exons were enumerated using featureCounts (subread v.1.4.4) requiring a minimum mapping quality of 10 using GRCm38.85 ensembl annotations (downloaded 08/26/2016). Counts were analyzed using the R package DESeq2 and libraries were normalized using the default median ratio method. Differentially expressed genes were required to pass a linear fold-change threshold of 1.5 and a FDR threshold of 0.1.

### ATAC-Seq

ATAC-seq was performed according to Buenrostro et al.^68^ with 50,000 sorted iNKT1 (CD27^+^, CD122^+^) or iNKT2 (CD27^+^, CD122^−^) cells. iNKT17 cells were not analyzed due to the low number of cells recovered from the SKG mice. Cells were lysed in 50 μl lysis buffer (10 mM TrisHCl, pH 7.4, 10 mM NaCl, 3 mM MgCl_2_, 0.1% (v/v) Molecular biology-grade IGEPAL CA-630) to extract nuclei. Nuclei were pelleted at 500×*g*. Nuclei were resuspended in 50 μl 1 × TD buffer containing 2.5 μl transposase (Nextera, Illumina). The transposase reaction was conducted for 40 min at 37 °C. DNA was purified using MinElute PCR Purification columns and kit (Qiagen) and barcoding were performed using Illumina compatible index primers (IDTdna.com). PCR was conducted for 11–12 cycles. Library purification was performed with the MinElute PCR Purification Kit (Qiagen) and library size selection was carried out using AMPureXP (Beckman Coulter). Libraries were quantified and size distribution was assessed using the Bioanalyzer High Sensitivity DNA Kit (Agilent). Paired-end sequencing was performed on a HiSeq 2500 (Illumina) with 50 cycles for each read. Raw data from the sequencer was demultiplexed and FASTQ files were generated using bcl2fastq conversion software (Illumina).

### ATAC-Seq analysis

Sequencing reads in FASTQ format were mapped to mouse genome (mm10) using bowtie (version 1.0.0) with parameters “-p 8 -m 1 --best --strata -X 2000 -S --fr --chunkmbs 1024.” Unmapped reads were processed with trim_galore using parameters “--paired --nextera --length 37 --stringency 3 –three_prime_clip_R1 1 --three_prime_clip_R2 1” before attempting to map again using the above parameters. These two bam files were merged and processed to remove reads mapping to the mitochondrial genome and duplicate reads (with picard MarkDuplicates). For visualization, genomic coverage for individual replicates were computed on 10 bp windows with MEDIPS using full fragments captured by ATAC-seq and used to generate average coverage with the Java Genomics Toolkit for each group. To identify peaks, the bam files containing unique, non-chrM reads were processed with samtools and awk using “‘{if(sqrt(/$9*/$9) < 100)print0,}’” to identify nucleosome free DNA fragments <100 nt in length. These subnucleosomal fragments were used to call peak summits for each replicate with MACS2 using parameters “--nomodel -q 0.00001 --keep-dup all --call-summits.” The summits for each peak from all replicates were expanded to regions with a uniform size of 500 bp. These regions from all replicates were merged into one global set of peaks and were filtered to remove peaks on the Y chromosome or those that overlapped ENCODE blacklisted regions. The number of transposase insertions within each region was computed for each replicate and these raw ATAC-seq counts per peak for all replicates were normalized using voom. Pairwise contrasts were performed with limma and differentially accessible regions were filtered based on an FDR adjusted *p*-value of < 0.01 and an estimated fold-change of at least 4. We computed the ATAC-seq density (number of transposase insertion sites per kilobase) and accessible regions were defined as those with a mean of 3 normalized insertions per kilobase. We used HOMER to identify motifs for transcription factor binding sites enriched in different groups of peaks.

### Statistical analysis

Groups were compared with two-tailed Student’s *t*-tests or one-way analysis of variance (ANOVA) with Prism 7 (Graphpad Software, Inc.). *p < 0.05, **p < 0.01, ***p < 0.001, and ****p < 0.0001.

### Data availability

Data that support the findings of this study have been deposited in NCBI GEO with the accession code GSE110327 (RNA-Seq) and GSE111427 (ATAC-Seq).

## Electronic supplementary material

Supplementary Information
