# Supplementary material for: TCR signal strength controls thymic differentiation of iNKT cell subsets
Source: Nat Commun. 2018 Jul 9;9:2650. doi: 10.1038/s41467-018-05026-6 (PMC6037704; doi:10.1038/s41467-018-05026-6)
Supplement: Supplementary file 1 — Supplementary Information [file 41467_2018_5026_MOESM1_ESM.pdf]

Supplementary Material

TCR signal strength controls thymic differentiation of iNKT cell subsets

Tuttle et al.

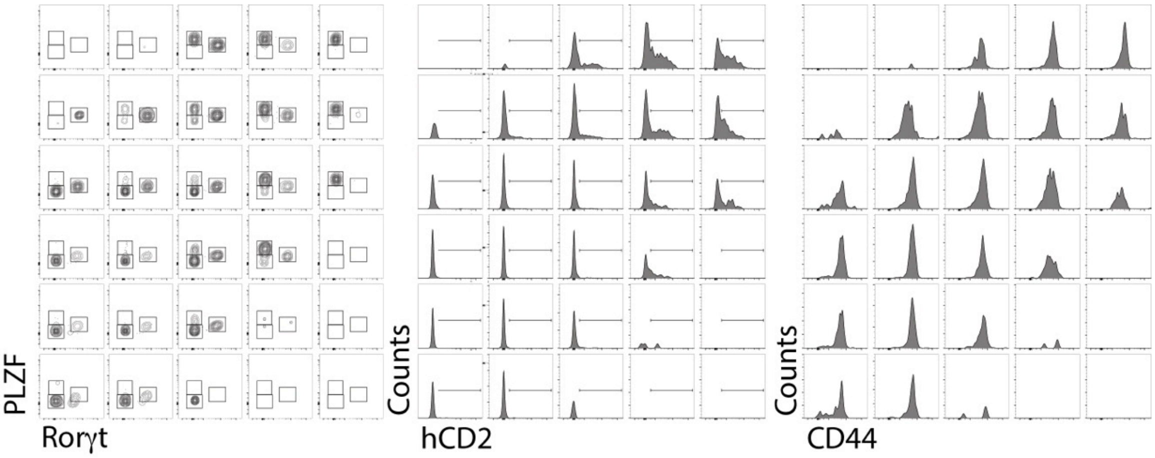

Supplementary Figure 1. Thymic iNKT subsets express TCRs of different avidities for the PBS57-CD1d tetramer. Cells from the thymus of BALB/c IL-4 reporter mice KN2 were stained with anti-TCRβ mAbs and PBS57-CD1d tetramers. iNKT cells were then electronically placed on a grid consisting of 30 gates (see Fig 1a). The proportion of NKT1 ($PLZF^{lo}$, $Ror\gamma t^{-}$, $Tbet^{+}$), NKT17 ($PLZF^{int}$, $Ror\gamma t^{+}$, $Tbet^{-}$), NKT2 ($PLZF^{hi}$, $Ror\gamma t^{-}$, $Tbet^{-}$) or $hCD2^{+}$ as well as the proportion of $CD44^{hi}$ cells in each gate was recorded.

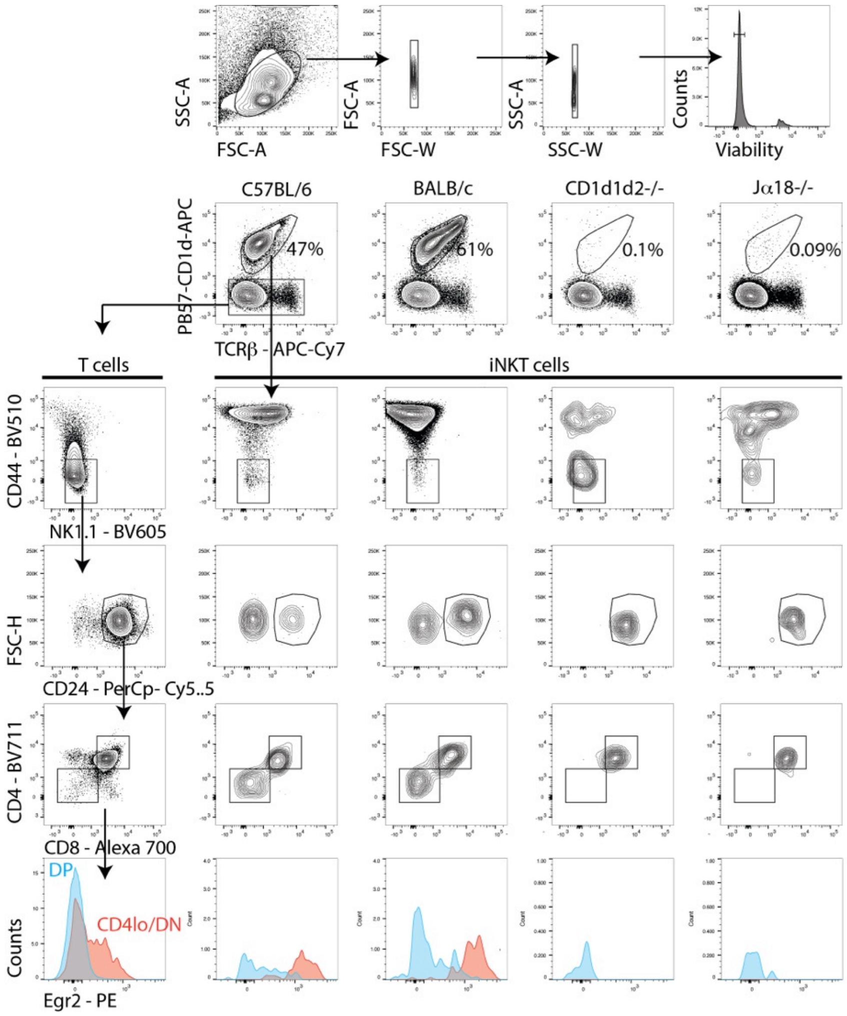

Supplementary Figure 2. Definition of stage 0 iNKT cells. Cells from the thymi of C57BL/6, BALB/c, CD1d1d2$^{-/-}$ and Jα18$^{-/-}$ mice were positively enriched for PBS57-CD1d tetramer^{+} cells using magnetic beads and an autoMACS Pro Separator and stained for the indicated markers. Doublets were excluded and only viable cells were included in the analysis. iNKT cells or "conventional" T cells were gated and analyzed following the gating strategy shown. CD44^{-} CD24^{+} iNKT cells contain some CD4^{+} CD8^{+} DP thymocytes that likely correspond to non-specific staining, as exemplified by the presence of this population in the thymi of CD1d1d2$^{-/-}$ and Jα18$^{-/-}$ mice. These DP cells do not express Egr2 or CD69 (Fig 5a). PBS57-CD1d tetramer^{+} TCRβ^{+} CD44^{-} CD24^{+} cells that are CD4^{lo} or CD4^{-}CD8^{-} (DN) are uniformly Egr2^{+} (and CD69^{+}) and can be detected only in the thymi of CD1d-sufficient mouse strains. Note that the levels of Egr2 expression detected in these cells is higher than what is detected in "conventional" T cells undergoing positive selection.

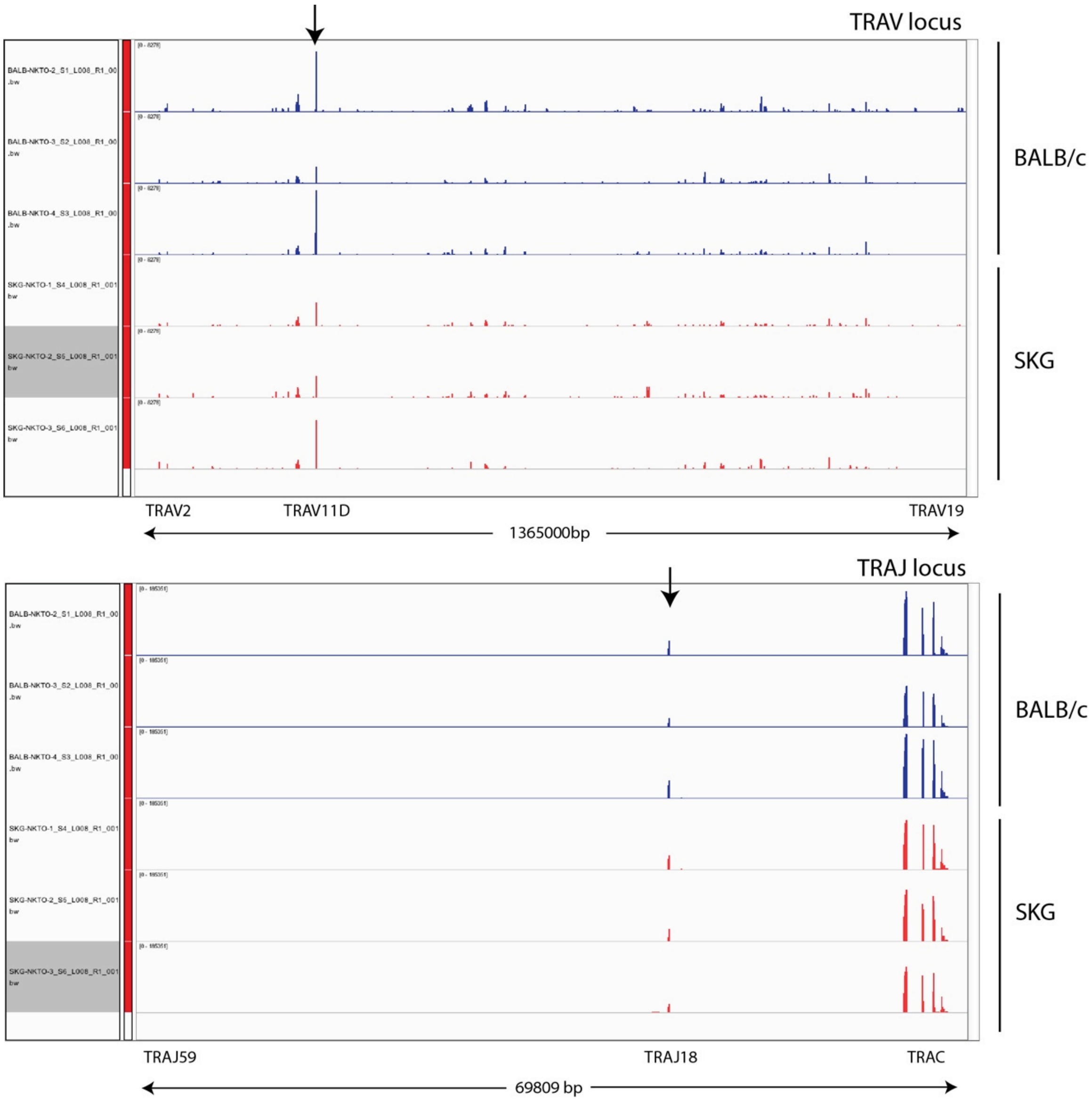

Supplementary Figure 3. Mapping of RNA-seq reads on the TRAV and TRAJ loci. Stage 0 iNKT cells from BALB/c and SKG mice were sorted and their RNA was extracted followed by library construction for Illumina sequencing. Three independent samples from BALB/c and SKG mice were prepared. Sequence reads were mapped to the mouse genome and individual tracks for each sample are shown for the whole TRAV (upper panel) and TRAJ (lower panel) loci. The results demonstrate a large enrichment for TRAV11D (encoding Vα14) and TRAJ18 (encoding Jα18) containing sequences in agreement with the TCR sequence expected from iNKT cells.

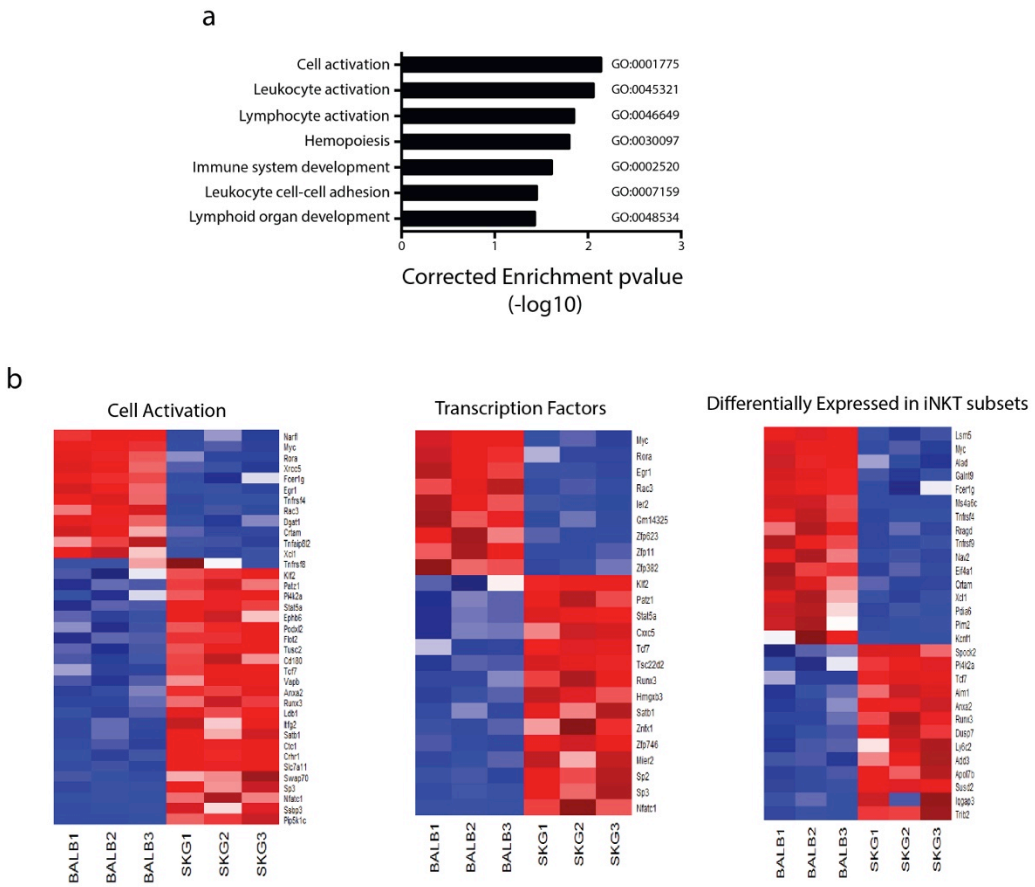

Supplementary Figure 4. Gene expression profile of thymic stage 0 iNKT cells from BALB/c and SKG mice. (**a**) Pathway-enrichment analysis of differentially expressed genes between stage 0 iNKT cells of BALB/c and SKG mice using gprofiler (http://biit.cs.ut.ee/gprofiler/) (**b**) Expression of genes encoding selected genes involved in cell activation (left), transcription factors (middle) and gene previously described as differentially expressed between iNKT cell subsets. The data are presented as row-wise z-scores (red, higher expression and blue, lower expression, relative to the other conditions). Data are from one experiment with three biological replicates per genotype.

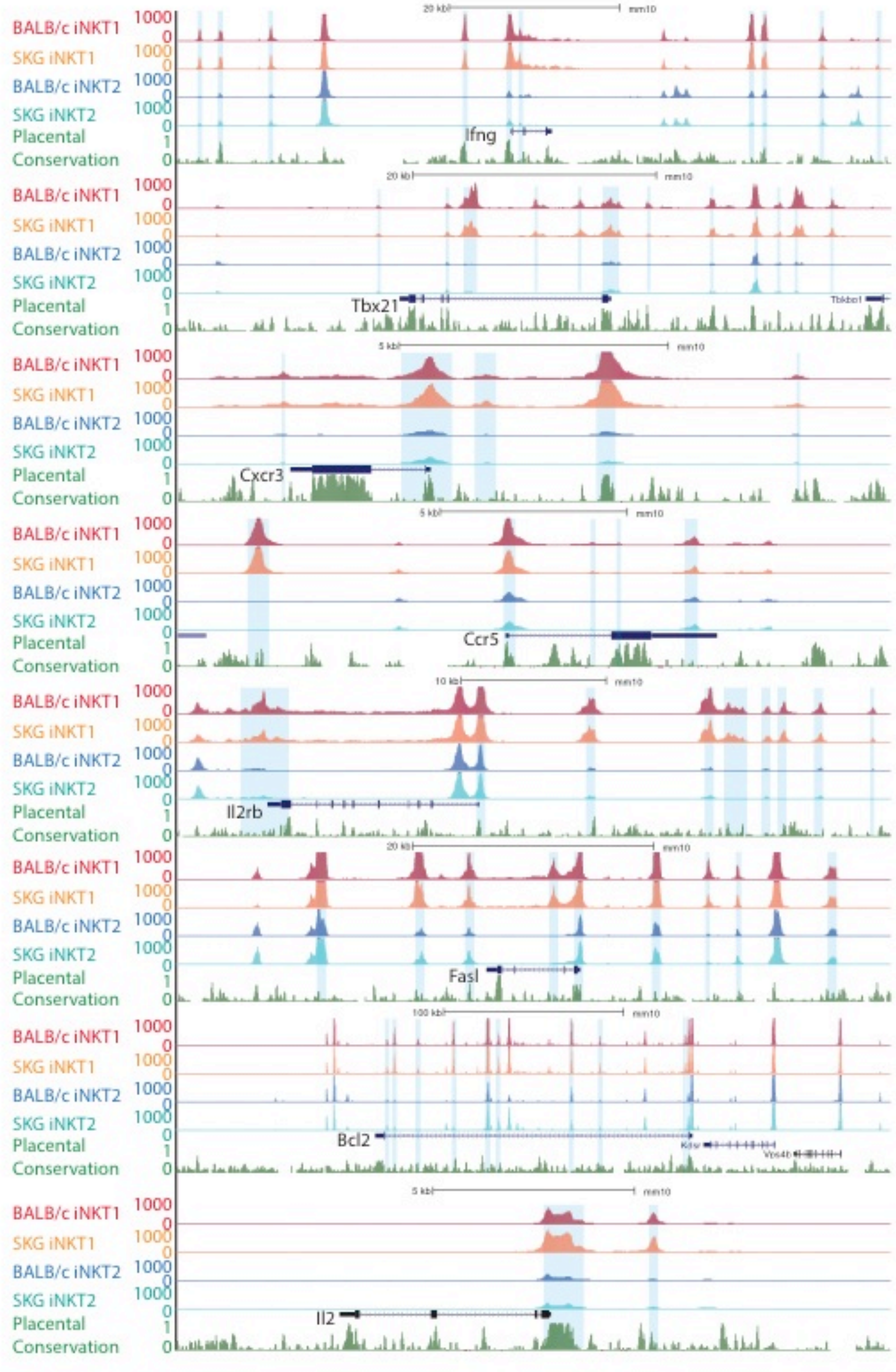

Supplementary Figure 5. Chromatin accessibility surrounding iNKT1 expressed genes. Mean ATAC sequencing coverage at the *Ifng*, *Tbx21*, *Cxcr3*, *Ccr5*, *IL2rb*, *Fasl*, *Bcl2* and *Il2* loci for BALB/c and SKG iNKT1 and iNKT2 cells with a scale from 0-1000 for the ATAC-Seq tracks. Placental conservation (in green) is indicated. Regions highlighted in light blue are more accessible in iNKT1 than in iNKT2 cells.

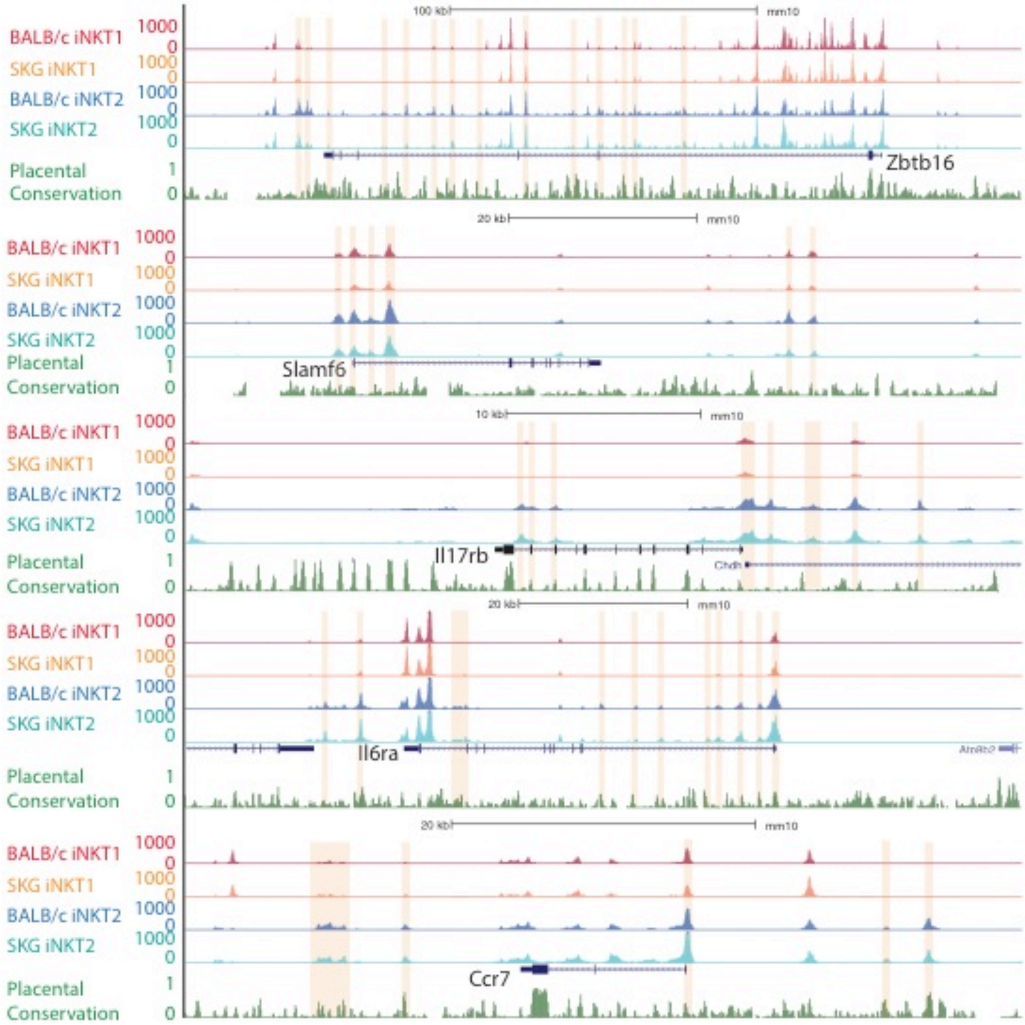

Supplementary Figure 6. Chromatin accessibility surrounding iNKT2 expressed genes. Mean ATAC sequencing coverage at the *Zbtb16*, *Slamf6*, *Il17rb*, *Il6ra* and *Ccr7* loci for BALB/c and SKG iNKT1 and iNKT2 cells with a scale from 0-1000 for the ATAC-Seq tracks. Placental conservation (in green) is indicated. Regions highlighted in light orange are more accessible in iNKT2 than in iNKT1 cells.

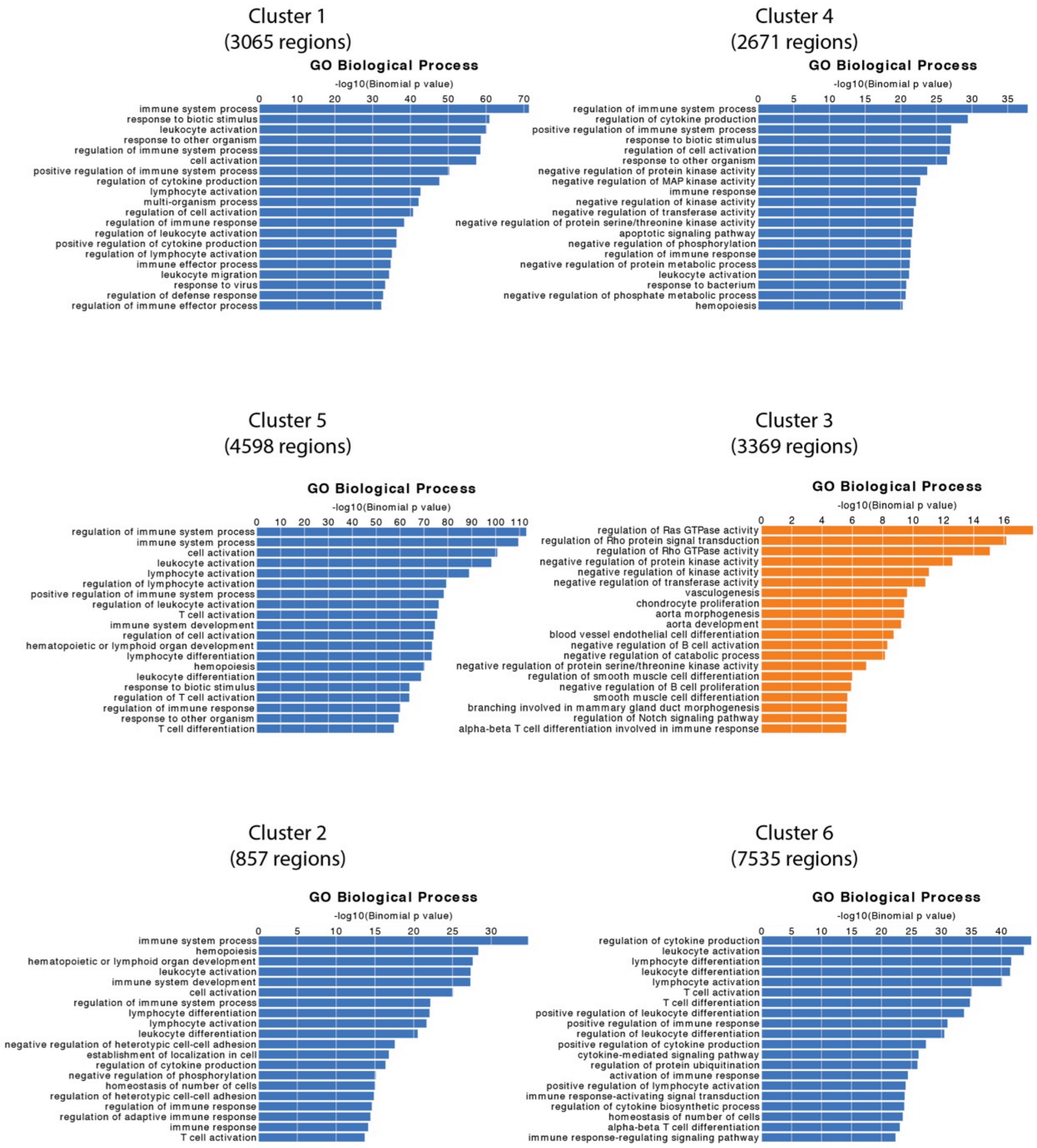

Supplementary Figure 7. Gene ontology (GO) terms enriched in peaks associated with each cluster defined in Fig 7f as determined through GREAT analysis. The number of regions in each cluster is indicated.

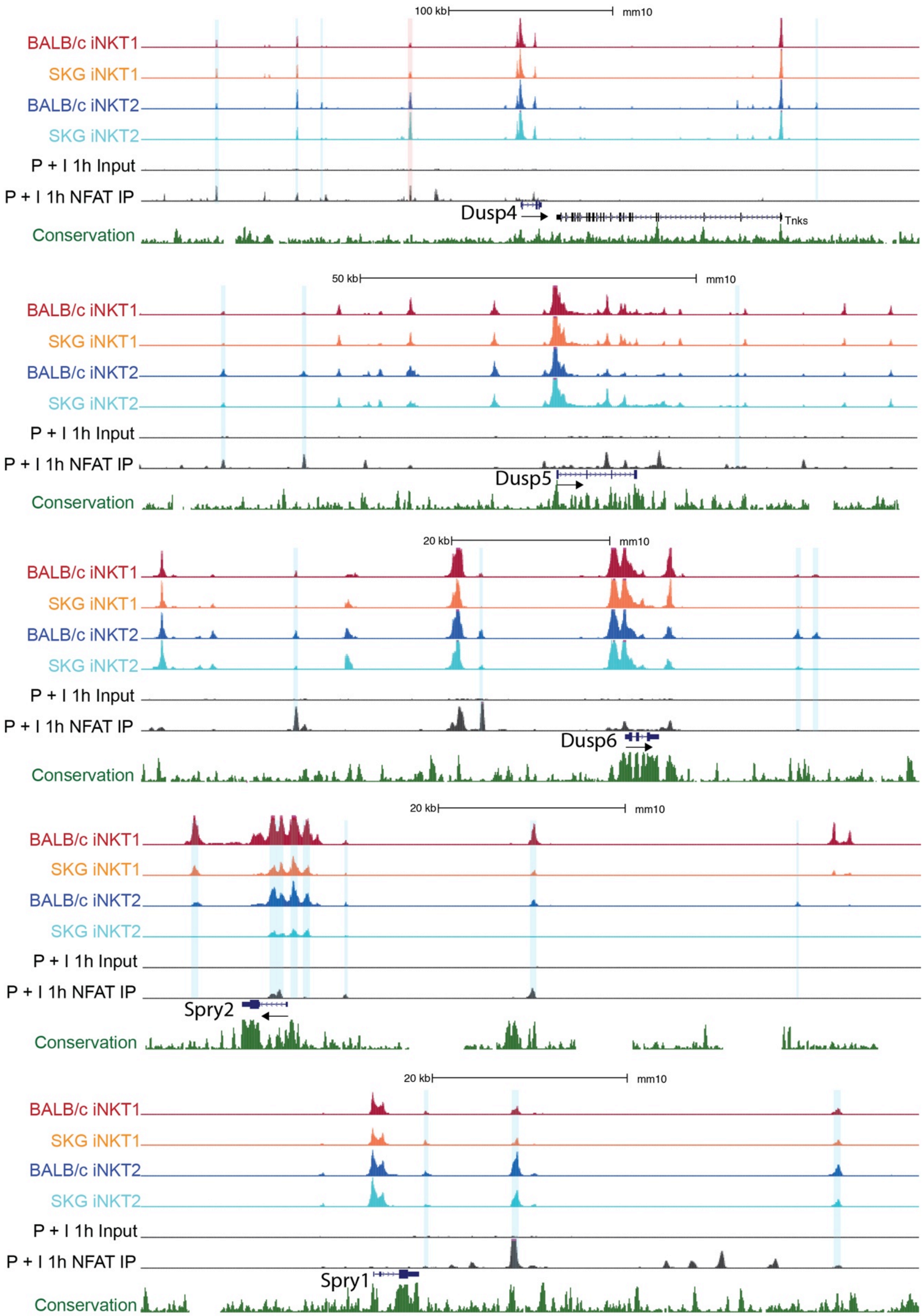

100 kb
mm10
BALB/c iNKT1
SKG iNKT1
BALB/c iNKT2
SKG iNKT2
P + I 1h Input
P + I 1h NFAT IP
Dusp4
Tnks
Conservation
50 kb
mm10
BALB/c iNKT1
SKG iNKT1
BALB/c iNKT2
SKG iNKT2
P + I 1h Input
P + I 1h NFAT IP
Dusp5
Conservation
20 kb
mm10
BALB/c iNKT1
SKG iNKT1
BALB/c iNKT2
SKG iNKT2
P + I 1h Input
P + I 1h NFAT IP
Dusp6
Conservation
20 kb
mm10
BALB/c iNKT1
SKG iNKT1
BALB/c iNKT2
SKG iNKT2
P + I 1h Input
P + I 1h NFAT IP
Spry2
Conservation
20 kb
mm10
BALB/c iNKT1
SKG iNKT1
BALB/c iNKT2
SKG iNKT2
P + I 1h Input
P + I 1h NFAT IP
Spry1
Conservation

Supplementary Figure 8. ATAC-seq signal profiles of genes identified by GREAT with the GO biological process of negative regulation of kinase activity. The profiles for *Dusp4*, *Dusp5*, *Dusp6*, *Spry2* and *Spry1* are shown for BALB/c iNKT1 (red), SKG iNKT1 (orange), BALB/c iNKT2 (Blue) and SKG iNKT2 (cyan). Peaks that are less accessible due to the SKG mutation are highlighted in blue while peaks that are more accessible in SKG cells over BALB/c cells are highlighted in orange. A scale from 0-1000 for the ATAC-Seq tracks was used. Tracks from NFAT ChIP in CD8 T cells stimulated in the presence or absence of PMA/ionomycin for 1 hour (ref [37]) are also included.

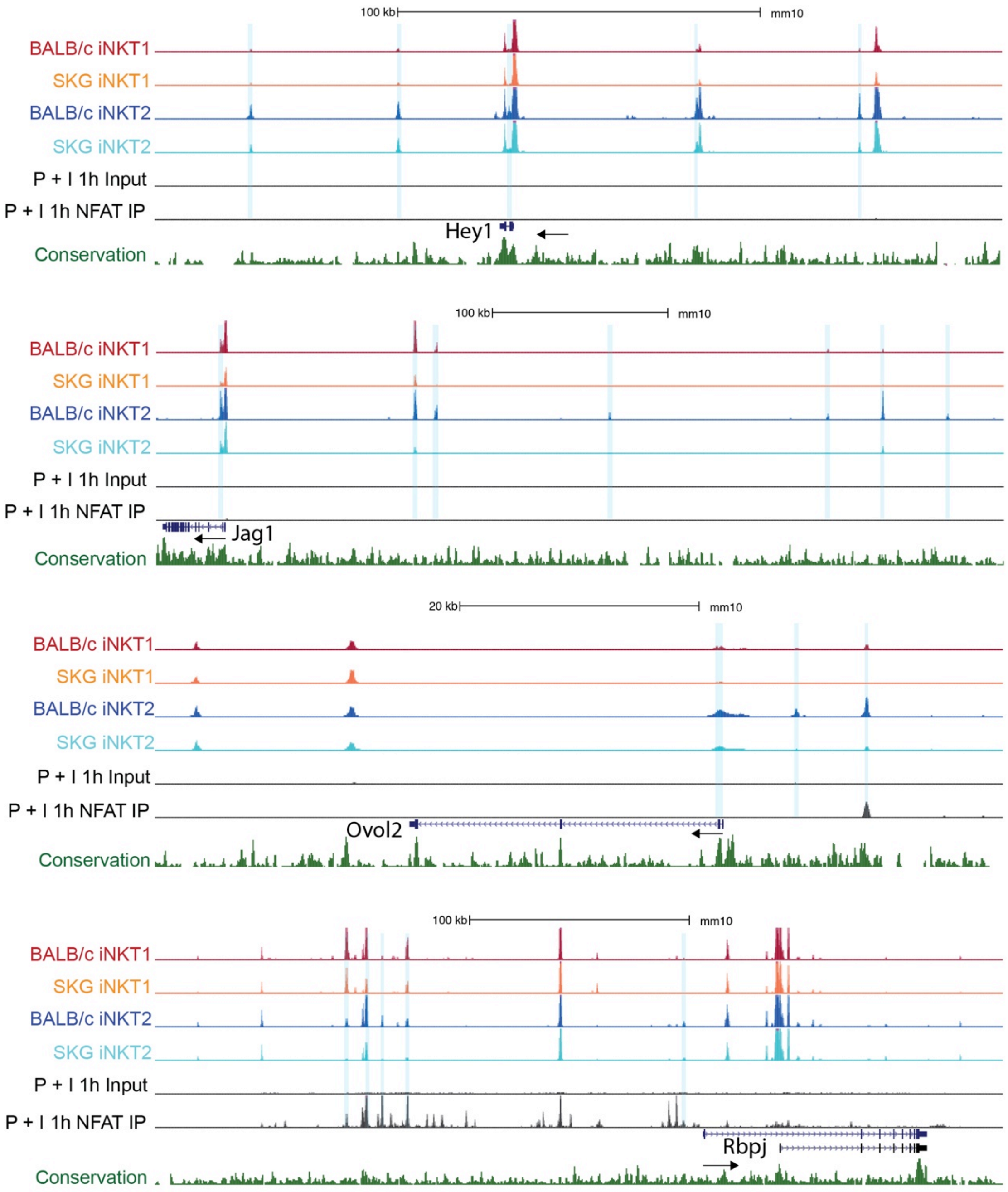

Supplementary Figure 9. ATAC-seq signal profiles of genes identified by GREAT with the GO biological process of regulation of NOTCH signaling pathway. The profiles for *Hey1*, *Jag1*, *Ovol2* and *Rbpj* are shown for BALB/c iNKT1 (red), SKG iNKT1 (orange), BALB/c iNKT2 (Blue) and SKG iNKT2 (cyan). Peaks that are less accessible due to the SKG mutation are highlighted in blue while peaks that are more accessible in SKG cells over BALB/c cells are highlighted in orange. A scale from 0-1000 for the ATAC-Seq tracks was used. Tracks from NFAT ChIP in CD8 T cells stimulated in the presence or absence of PMA/ionomycin for 1 hour (ref [37]) are also included.

Regulation of Ras GTPase activity	regulation of Rho protein signal transduction	regulation of Rho GTPase activity	negative regulation of protein kinase activity	negative regulation of kinase activity	negative regulation of transferase activity	vasculogenesis
Adcyap1	Als2	Als2	Adar	Adar	Adar	Ccm2
Agap1	Apoa1	Arhgap10	Adarb1	Adarb1	Adarb1	Cited2
Agfg1	Arhgap10	Arhgap15	Apc	Apc	Apc	Ctnnb1
Als2	Arhgap15	Arhgap24	Bmp7	Bmp7	Bmp7	Epha2
Arap2	Arhgap24	Chn1	Casp3	Casp3	Casp3	Fbxw7
Arhgap10	Arhgef18	Dlc1	Cdkn2a	Cdkn2a	Cdkn2a	Fgf1
Arhgap15	Arhgef26	Dock4	Dus2l	Dus2l	Cln8	Fgfr2
Arhgap24	Arhgef28	Efna5	Dusp16	Dusp16	Dus2l	Gata4
Asap1	Chn1	Epha1	Dusp4	Dusp4	Dusp16	Hdac7
Asap2	Cul3	Epha2	Dusp5	Dusp5	Dusp4	Hey1
Chn1	Dlc1	Epha3	Dusp6	Dusp6	Dusp5	Hhex
Dgki	Dock4	Ephb3	Epha1	Epha1	Dusp6	Kdr
Dlc1	Efna5	Fzd10	Fbxo7	Fbxo7	Epha1	Myo1e
Dock4	Epha1	Gpr65	Gadd45a	Gadd45a	Fbxo7	Ntrk2
Efna5	Epha2	Itgb1bp1	Gadd45g	Gadd45g	Gadd45a	Pdgfrb
Epha1	Epha3	Jun	Gnb2l1	Gnb2l1	Gadd45g	Ptprj
Epha2	Ephb3	Ntrk2	Hmgcr	Hmgcr	Gnb2l1	Rasa1
Epha3	Farp1	Ophn1	Ibtk	Ibtk	Hmgcr	Rasip1
Ephb3	Fgd5	Prex2	Igf1r	Igf1r	Ibtk	Sgpl1
Fzd10	Fzd10	Ralbp1	Itgb1bp1	Itgb1bp1	Igf1r	Shh
Gdi2	Gpr65	Rasgrp1	Itprip	Itprip	Itgb1bp1	Sox17
Gnas	Itgb1bp1	Rasip1	Kat2b	Kat2b	Itprip	T
Gpr65	Jun	Sod1	Lats2	Lats2	Kat2b	Tgfbr2
Itgb1bp1	Lpar1	Srgap1	Lax1	Lax1	Lats2	Tgfbr3
Jun	Ntrk2	Tbc1d7	Pkia	Pkia	Lax1	Tmem100
Ntrk2	Ophn1	Tiam1	Pkib	Pkib	Pkia	Wars2
Ntrk3	Plekhg1		Prkag2	Prkag2	Pkib	Wnt7a
Ophn1	Plekhg3		Prkca	Prkca	Prkag2	Wnt7b
Prex2	Plekhg6		Prkcd	Prkcd	Prkca	Wt1
Ralbp1	Prex2		Prkrip1	Prkrip1	Prkcd	Zfp36l1
Ralgapa2	Ralbp1		Ptpn6	Ptpn6	Prkrip1	Zmiz1
Rap1a	Rasgrf2		Ptprj	Ptprj	Ptpn6	
Rap1gap	Rasgrp1		Sfrp5	Sfrp5	Ptprj	
Rasa1	Rasip1		Socs5	Socs5	Sfrp5	
Rasa2	Sod1		Spred2	Spred2	Socs5	
Rasgrp1	Spata13		Spry1	Spry1	Spred2	
Rasgrp3	Srgap1		Spry2	Spry2	Spry1	
Rasip1	Tbc1d7		Trib1	Trib1	Spry2	
S1pr1	Tiam1		Trib2	Trib2	Trib1	
Sgsm2	Tiam2		Ubash3b	Ubash3b	Trib2	
Sipa1l1	Trio		Uchl1	Uchl1	Ubash3b	
Sod1	Vav3		Zfyve28	Zfyve28	Uchl1	
Spry1					Zfyve28	
Spry2						
Srgap1						
Tbc1d1						
Tbc1d14						
Tbc1d2						
Tbc1d22a						
Tbc1d23						
Tbc1d4						
Tbc1d7						
Tbck						
Tiam1						
Usp6nl						

chondrocyte proliferation	aorta morphogenesis	aorta development	blood vessel endothelial cell differentiation	negative regulation of B cell activation	negative regulation of catabolic process	negative regulation of protein serine/threonine kinase activity	regulation of smooth muscle cell differentiation	negative regulation of B cell proliferation	smooth muscle cell differentiation	branching involved in mammary gland duct morphogenesis	regulation of Notch signaling pathway	alpha-beta T cell differentiation involved in immune response
Ctgf	Eya1	Eya1	Ccm2	Btla	Adra2a	Apc	Ereg	Btla	Acvr1	Ar	Aak1	Eomes
Ddr2	Hes1	Hes1	Hey1	Casp3	Cidea	Bmp7	Fgfr2	Casp3	Ctnnb1	Areg	Bmp7	Gadd45g
Hmga2	Hey1	Hey1	Kdr	Cdkn2a	Cnr1	Casp3	Prdm6	Cdkn2a	Ednrb	Csf1	Dlx2	Gata3
Ihh	Jag1	Jag1	Rbpj	Ctla4	Cpeb2	Cdkn2a	Shh	Ctla4	Gata6	Ddr1	Eya1	Ifng
	Mylk	Mylk	Tmem100	Fcgr2b	Fhit	Dusp16	Tmsb4x	Fcgr2b	Hes1	Epha2	Fbxw7	Irf4
	Pdgfrb	Pdgfrb		Hmgb3	Fyn	Dusp4	Tshz3	Il10	Itga8	Esr1	Hes1	Lef1
	Prox1	Prox1		Id2	Grin2a	Dusp5	Zeb1	Prdm1	Mef2c	Phb2	Il6st	Ptger4
	Rbpj	Rbpj		Il10	Hdac4	Dusp6			Mkl2	Slc12a2	Itgb1bp1	Stat6
	Sox4	Sox4		Ndfip1	Ier3	Fbxo7			Npnt	Tbx3	Jag1	
	Srf	Srf		Prdm1	Il10	Hmgcr			Pdgfrb	Tfap2c	Neurl1a	
				Samsn1	Il3	Kat2b			Smad6	Tram1	Ovol2	
					Lrpprc	Lats2			Srf	Vdr	Rbpj	
					Mad2l1	Lax1			Tbx18		Slc35c1	
					Mdm4	Prkca			Tshz3		Trp63	
					Npc1	Prkcd			Wnt7b			
					Pde3b	Ptpn6						
					Prkaa1	Ptprj						
					Ptpn3	Sfrp5						
					Rasip1	Spred2						
					Shh	Spry1						
					Smad3	Spry2						
					Tbc1d14	Uchl1						
					Timp3							
					Wac							

Supplementary Table I. List of genes associated with each GO biological terms for cluster 3.
